# Ultrasound-Responsive Nanoparticles Enable Hydrophobic Antibiotic Release and Deep Penetration for Biofilm Treatment

**DOI:** 10.1021/jacsau.5c01711

**Published:** 2026-02-25

**Authors:** Maria L. Odyniec, Daniel J. Bell, Benjamin M. Gallant, Rininta Firdaus, Grace Ball, Liam Hughes, Rebecca Oxtoby, Benjamin J. Hewitt, Christopher M. Williams, Asier R. Muguruza, Tim W. Overton, Hung-Ji Tsai, Yu-Lung Chiu, Dominik J. Kubicki, A. Damien Walmsley, Sarah A. Kuehne, Zoe Pikramenou

**Affiliations:** † School of Chemistry, College of Engineering and Physical Sciences, 1724 University of Birmingham, Edgbaston, Birmingham B15 2TT, U.K.; ‡ School of Dentistry, College of Medical and Dental Sciences, University of Birmingham, Edgbaston, Birmingham B15 2TT, U.K.; § School of Metallurgy and Materials, College of Engineering and Physical Sciences, University of Birmingham, Edgbaston, Birmingham B15 2TT, U.K.; ∥ School of Biosciences, Institute of Microbiology and Infection, University of Birmingham, Edgbaston, Birmingham B15 2TT, U.K.; ⊥ School of Biomedical Sciences, College of Medicine and Health, University of Birmingham, Edgbaston, Birmingham B15 2TT, U.K.; # School of Chemical Engineering, College of Engineering and Physical Sciences, University of Birmingham, Edgbaston, Birmingham B15 2TT, U.K.; ∇ School of Science and Technology, 6122 Nottingham Trent University, Nottingham NG1 4FQ, U.K.

**Keywords:** ultrasound, silica, nanoparticles, antibiotic release, hydrophobic antibiotic, core@shell

## Abstract

Localized delivery of antibiotics is a promising strategy that leads to transformative treatment pathways of bacterial biofilms and increases the effectiveness of their administration in contrast to traditional delivery methods requiring high antibiotic doses. Hydrophobic antibiotics have poor activity against bacterial biofilms due to their limited penetration and are particularly challenging to deliver. Nanoparticles are ideal drug delivery agents to achieve spatially controlled delivery, but commonly their designs are either soft or porous, which limits temporally triggered release, with the result that most of the antibiotic does not reach deeply into the biofilm. In this study, we present designs of nonporous silica nanoparticles that encapsulate a lipophilic antibiotic, rifampicin, with noncovalent interactions and enable controlled release triggered by Low-Frequency Ultrasound (LFUS). *Staphylococcus aureus* biofilms treated with the nonporous, core@shell, rifampicin-encapsulated nanoparticles, **RIF⊂PhSiO**_**2**_ **@SiO**_**2**_, combined with LFUS, achieved 90% biofilm eradication, compared to 20% without ultrasound; treatment with free rifampicin and LFUS resulted only in a 10% reduction. Nanoparticle penetration into biofilm layers was visualized using fluorescent nanoparticles prepared with coencapsulation of the Nile red fluorophore, **RIF+NR⊂PhSiO**_**2**_ **@SiO**_**2**_. Confocal fluorescence imaging of the biofilms demonstrated penetration of the nanoparticles throughout all the layers of the biofilm upon LFUS application, in sharp contrast to their presence in only the top few biofilm layers without LFUS. Scanning Electron Microscopy of the biofilms confirmed the presence of nanoparticles and the dual role of LFUS in promoting penetration and facilitating drug release by disrupting molecular interactions within the nanoparticle. This work introduces a design paradigm for nonporous nanoparticle agents combined with ultrasound, enabling both temporal and spatial control of drug release in bacterial biofilms. This will open transformative therapeutic approaches for effective localized delivery of drugs that have previously been challenging to deliver.

## Graphical abstract

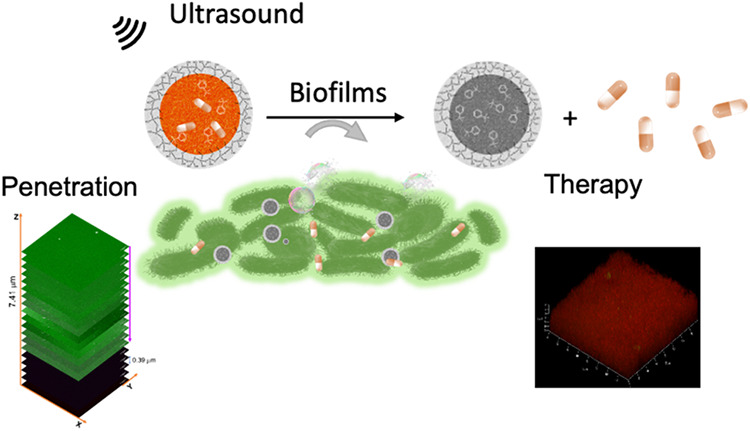

## 1 Introduction

Effective localized delivery of antibiotics is particularly desirable due to the large quantities of antibiotics commonly administered systemically to eradicate infections, greatly increasing the risk of antimicrobial resistance.^1^ Biofilms, surface-associated communities of bacteria enveloped in a matrix of self-produced extracellular polymeric substances (EPS), are responsible for 80% of infections and are difficult to treat, especially with hydrophobic antibiotics, when penetration into a hydrated environment is often restricted even with formulations of antibiotics. Many challenging-to-deliver antibiotics can be repurposed by efficient delivery methods. Nanoparticles are ideal drug carriers for localized delivery, increasing the drug dose on a targeted site, assisted by their high surface area to volume ratio.^2^ Increased penetration of drugs into biofilms has been reported in various ways, including encapsulation within lipid-coated and polymer nanoparticles.^3,4^ However, temporal control of delivery is important to eliminate “burst” drug release before it reaches all the layers of biofilm. Additionally, recent studies have highlighted the lack of antimicrobial action against persister and other antibiotic-recalcitrant cells^5^ present deep within biofilms in difficult-to-reach pockets.^6,7^

The design of nanosized delivery systems is critical to inducing control of drug release from porous nanosized networks. Synthetic modifications of soft nanoparticles (micelles, polymers) have been introduced to allow drug release triggered by physicochemical stimuli.^8,9^ Silica nanoparticles are attractive inorganic carriers with high biocompatibility, featuring a hard core and a surface that can be easily modulated with chemical and biochemical inputs.^10–14^ Mesoporous silica nanoparticles have dominated the applications for drug delivery due to the high surface area, which enables the adsorption of large amounts of drugs in porous channels up to 50 nm. However, uncontrolled drug release from the porous channels takes place, with much of the drug being delivered to nontargeted areas, and approaches have been used to cap the pore channels and modulate the release with chemical and biochemical inputs.^10,14^

Temporal control of drug release can be envisaged with the application of an external stimulus. Low-frequency ultrasound (LFUS), (20–40 kHz) is an emerging technique with clinical use in dental and wound treatments.^15,16^ LFUS has been reported to disrupt biofilm structures and reactivate persister cells.^17–21^ Application of LFUS exposes drug delivery systems to mechanical, thermal, and chemical effects, which can enhance the release kinetics of drugs from delivery carriers.^22–24^ This is in contrast with high frequency ultrasound (>1 MHz), which is used in covalent bond cleavage.^25^

We have previously demonstrated that LFUS may trigger the release of antibacterial agents from mesoporous silica nanoparticles.^26,27^ However, a direct comparison showed that nonporous, amorphous silica nanoparticles exhibited a markedly stronger cavitation-mediated release, mainly due to the mechanical forces of the bubbles formed in solution. We therefore selected the nonporous design for subsequent work.^28^

We report herein a core@shell silica nanoparticle design with a hydrophobic core based on organosilica precursors and a hydrophilic shell for better biocompatibility with biofilms. To avoid the “burst” release of the antibiotic from mesoporous nanoparticles, we have examined the encapsulation of the antibiotic during silica growth, forming nonporous nanoparticles, and demonstrate the importance of the hydrophobicity of the internal silica framework for the encapsulation and release of lipophilic antibiotics. Additionally, our approach allows the coencapsulation of a fluorophore in order to visualize the penetration of the nanoparticles through the biofilm. The challenges of studying antibiotic penetration though biofilms are well-known^29^ and most studies have relied on staining the bacterial or the biofilm matrix, where the signal vanishes upon antibiotic-induced lysis and thus provides only an indirect approximation of penetration depth.^30,31^

We have chosen rifampicin, a lipophilic antimicrobial with challenges in effective, localized drug delivery.^32–34^ It is widely used to treat tuberculosis, as well as *Staphylococcus aureus* ^35^ and orthopedic device-associated infections.^33,34^ *S. aureus* is selected as a model organism due to its well-documented role in wound and chronic infection-associated biofilms. Although planktonic *S. aureus* is generally susceptible to rifampicin, its biofilm form presents a significant therapeutic challenge, exhibiting increased resistance to antimicrobial agents. The decreased efficacy of rifampicin delivery in biofilm-mediated infections contributes to long-term or high-dose regimes, which can lead to rifampicin resistance. Previous delivery approaches involved mesoporous nanoparticles.^36,37^ Efficient rifampicin delivery systems for the eradication of biofilms and associated persister or otherwise recalcitrant bacteria are required to reduce drug loading and resistance development.

To study the hydrophobicity requirements for rifampicin encapsulation within the silica framework, we have used two designs: core@shell particles with a hydrophobic core based on organosilica precursors and a hydrophilic shell and a design based on co-condensation of phenyl-substituted silane and nonsubstituted ethoxysilane precursors to increase framework hydrophilicity. Both approaches involve encapsulation of rifampicin with either the two-step approach, core@shell particles, **RIF⊂PhSiO**_**2**_ **@SiO**_**2**_, or one-pot synthesis to yield **RIF⊂PhSiO**_**2**_ **·SiO**_**2**_ (Figure 1). Silica framework modification with organosilica precursors^38–41^ has been previously used, although most designs have focused on external surface hydrophobicity.^42–44^ Electron microscopy and solid-state nuclear magnetic resonance (ssNMR) have been employed to elucidate the differences in the inner silica structural framework. We investigate the release of rifampicin *in vitro* and the nanoparticle activity against planktonic and single-species biofilms of *S. aureus* for controlled, localized, triggered delivery upon application of LFUS. We also report that coencapsulation of Nile red with rifampicin leads to fluorescent nanoparticles **RIF+NR⊂PhSiO**_**2**_ **@SiO**_**2**_ that allow evaluation of their penetration in the biofilms. The studies show the importance of core@shell nanoparticle designs, responsive to LFUS for rifampicin release in effective biofilm eradication, contrasted with rifampicin on its own or the absence of LFUS.

**Figure 1:**
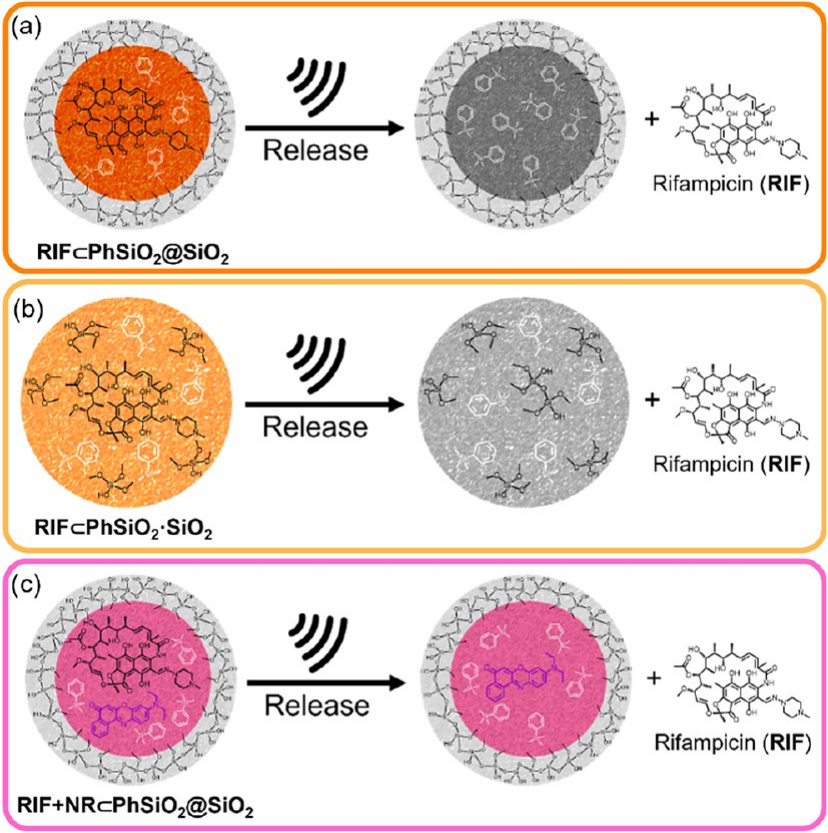
Designs of ORMOSIL nanoparticles for LFUS-induced release of rifampicin: (a) core@shell **RIF⊂PhSiO**_**2**_ **@SiO**_**2**_, (b) co-condensation **RIF⊂PhSiO**_**2**_ **·SiO**_**2**_, and (c) fluorescent core@shell **RIF+NR⊂PhSiO**_**2**_ **@SiO**_**2**_.

## 2 Results and Discussion

### 2.1 Synthesis and Characterization of Rifampicin-Encapsulated ORMOSIL Nanoparticles

To introduce hydrophobicity into the silica nanoparticle framework, an aromatic alkoxysilane, trimethoxyphenylsilane, PhTMS, was used as a building block for the incorporation of hydrophobic moieties (PhSiO_2_) into the silica framework, increasing the favorability of rifampicin encapsulation compared to a plain silica framework. Two different ORMOSIL nanoparticle designs were prepared with encapsulated rifampicin (Figure 2): core@shell^45^ featuring a PhSiO_2_ core with rifampicin included during its growth and a SiO_2_ shell, **RIF⊂ PhSiO**_**2**_ **@SiO**_**2**_, to impart a hydrophilic nature to the nanoparticle surface limiting flocculation and thereby increasing stability in aqueous biological media and particles based on co-condensation of both agents in the presence of rifampicin, yielding a silica framework with PhSiO_2_ and SiO_2_ moieties distributed randomly throughout the nanoparticle, **RIF⊂PhSiO**_**2**_ **·SiO**_**2**_. Fluorescent particles were prepared by the addition of the silica precursors in a solution of both agents so that the silica growth took place with the cargo molecules present. Nanoparticles without any encapsulated guests for each method **PhSiO**_**2**_ **@SiO**_**2**_ and **PhSiO**_**2**_ **·SiO**_**2**_ were also synthesized and characterized for comparison. FT-IR analysis of the nanoparticles is not sensitive enough to provide identification of the core@shell and co-condensed nanoparticles (Figure S6).

**Figure 2:**
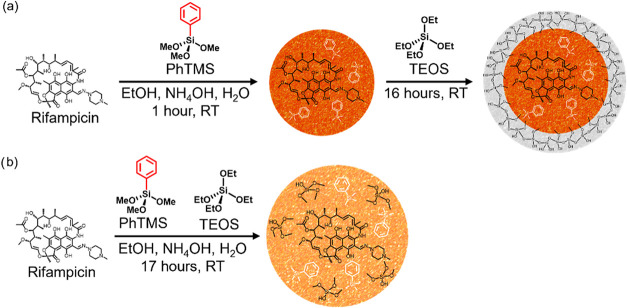
Two synthetic approaches for (a) core@shell **RIF⊂PhSiO**_**2**_ **@SiO**_**2**_ and (b) co-condensed assembly **RIF⊂PhSiO**_**2**_ **·SiO**_**2**_.

The rifampicin-encapsulated nanoparticles were characterized by an array of techniques. The morphology and size of the nanoparticles were investigated by transmission electron microscopy (TEM) and dynamic light scattering (DLS) in water (Figure 3). Following the first step of the core@shell synthesis, TEM analysis showed the core particles formed with an average diameter of 35 ± 4 nm, and the final core@shell particles, **RIF⊂PhSiO**_**2**_ **@SiO**_**2**_, have an average diameter of 90 ± 25 nm (polydispersity index, PDI = 0.11, *n* = 50) by TEM, with the hydrodynamic diameter (by volume) being in reasonable agreement (100 ± 30 nm, PDI = 0.26). The co-condensation nanoparticles, **RIF⊂PhSiO**_**2**_ **·SiO**_**2**_, have an average diameter of 54 ± 6 nm (PDI = 0.01, *n* = 50) by TEM (Figure 3). There is also a difference in the ζ-potential of the nanoparticles in water, which varies from −46 ± 7 mV for core@shell particles **RIF⊂PhSiO**_**2**_ **@SiO**_**2**_ to −27 ± 5 mV for **RIF⊂PhSiO**_**2**_ **·SiO_2_**. This is attributed to the presence of the Si–OH groups dominating the nanoparticle surface in the core@shell particles^46^ in contrast with the presence of the phenyl group in the co-condensation particles.

**Figure 3:**
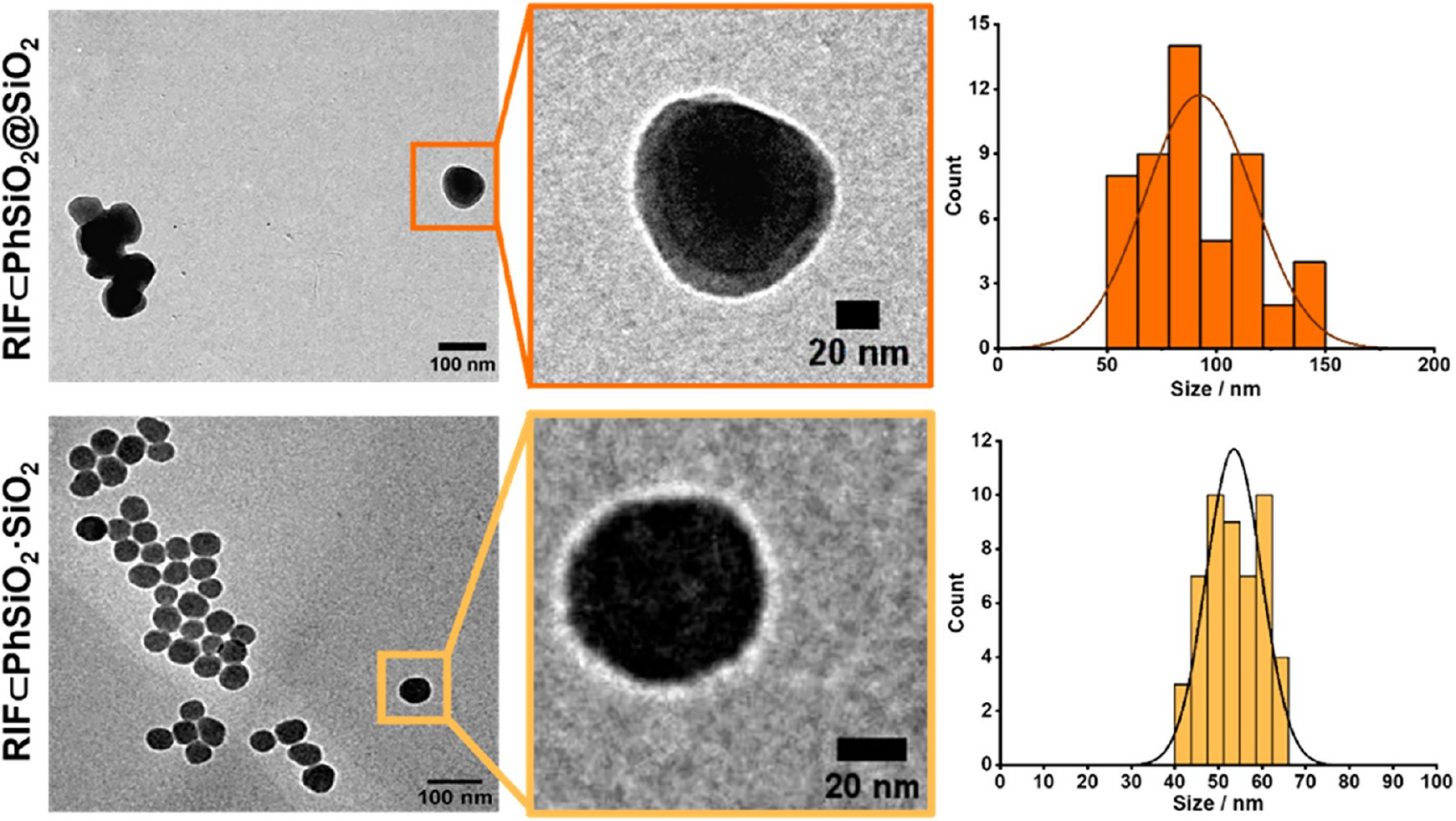
Transmission electron microscopy images of **RIF⊂PhSiO**_**2**_ **@SiO**_**2**_ (top) and **RIF⊂PhSiO**_**2**_ **·SiO**_**2**_ (bottom) nanoparticles, with size distribution histograms (*n* = 50).

Optical spectroscopy confirms the presence of rifampicin in **RIF⊂PhSiO**_**2**_ **@SiO**_**2**_ and **RIF⊂PhSiO**_**2**_ **·SiO**_**2**_. UV–vis spectra (reflectance mode) of nanoparticle powders show the characteristic rifampicin absorption bands with λ_max_ at 340 and 480 nm (Figure S2) arising from π–π* transitions. The intense peak at 260 nm corresponds to the phenyl moiety within the organosilica framework. The uptake of rifampicin into the nanoparticles was quantified by DMSO elutions.^36^ Nanoparticles were dispersed in DMSO at a concentration of 2 mg/mL for 16 h and subjected to centrifugation after a clear colorless suspension had formed; the resulting supernatant was then analyzed by UV–vis spectroscopy. Rifampicin uptake into **RIF⊂PhSiO**_**2**_ **@SiO**_**2**_ (1.45 ± 0.20 μg_**RIF**_/mg_NP_, 0.145 ± 0.020 wt %) was 1.7 times higher than the uptake into **RIF⊂PhSiO**_**2**_ **·SiO**_**2**_ (0.63 ± 0.05 μg_**RIF**_/mg_NP_, 0.063 ± 0.005 wt %). Total elution of rifampicin from the nanoparticles was confirmed by solid-state UV–vis spectroscopy with the absence of any rifampicin peaks. Fluorescent particles were prepared in order to evaluate the penetration of the nanoparticles within biofilms by coencapsulating Nile red together with rifampicin, yielding **RIF+NR⊂PhSiO**_**2**_ **@SiO**_**2**_. Nile red was chosen for encapsulation due to its hydrophobicity and because it can be readily visualized by confocal laser scanning microscopy. The final **RIF+NR⊂PhSiO**_**2**_ **@SiO**_**2**_ nanoparticles displayed an average diameter of 90 ± 13 nm (PDI = 0.02, *n* = 50) by TEM (Figure S1). Their excitation and emission properties show characteristic λ_exc_ at 564 nm and emission maxima λ_em_, at 622 nm (Figure S3) with a photoluminescence quantum yield of 45 ± 14%, which double in value to the one observed in mesoporous silica nanoparticles^47^ or to the dye in polar solvents.^48^ The surface area of the nanoparticles was measured via nitrogen porosimetry (Figure S7). The BET surface area of the plain **PhSiO**_**2**_ **@SiO**_**2**_ nanoparticles was over 8 times lower than the surface area of MCM-41 type mesoporous silica nanoparticles, as expected due to their amorphous nature. The particles with encapsulated rifampicin showed a significant surface area decrease.

Energy-dispersed X-ray (EDX) spectroscopy and scanning transmission electron microscopy (STEM) were used to further characterize the samples. High-angle annular dark field scanning transmission electron microscopy (HAADF-STEM) shows that the surface of the **RIF⊂PhSiO**_**2**_ **@SiO**_**2**_ particles is not smooth and there is a nonuniform dark-light patterning (Figure 4), indicative of small nonperiodic pores on the surface of the nanoparticle. EDX mapping of the core@shell particles **RIF⊂PhSiO**_**2**_ **@SiO**_**2**_ shows a distinct core area where Si and C signals overlap and a shell with no C signal, consistent with the expected core@shell design. Intensity plots for Si and C, measured through cross sections of the nanoparticles, indicated the shell was 1–4 nm thick, calculated by the different depths at which each signal trace returned to its baseline value, with areas with only the Si signal highlighted in purple (Figure 4). The oxygen signal is observed throughout the particle (Figure S4). HAADF-STEM of the co-condensation nanoparticles, **RIF⊂PhSiO**_**2**_ **·SiO_2_**, reveals a smoother surface with no patterning and EDX indicated that Si and C distributions do not present a shell but are distributed throughout the particle (Figure 5) similarly with oxygen (Figure S4) as expected. Nitrogen mapping via EDX could not be employed due to the relatively low concentration of N as compared with the rest of the material.

**Figure 4:**
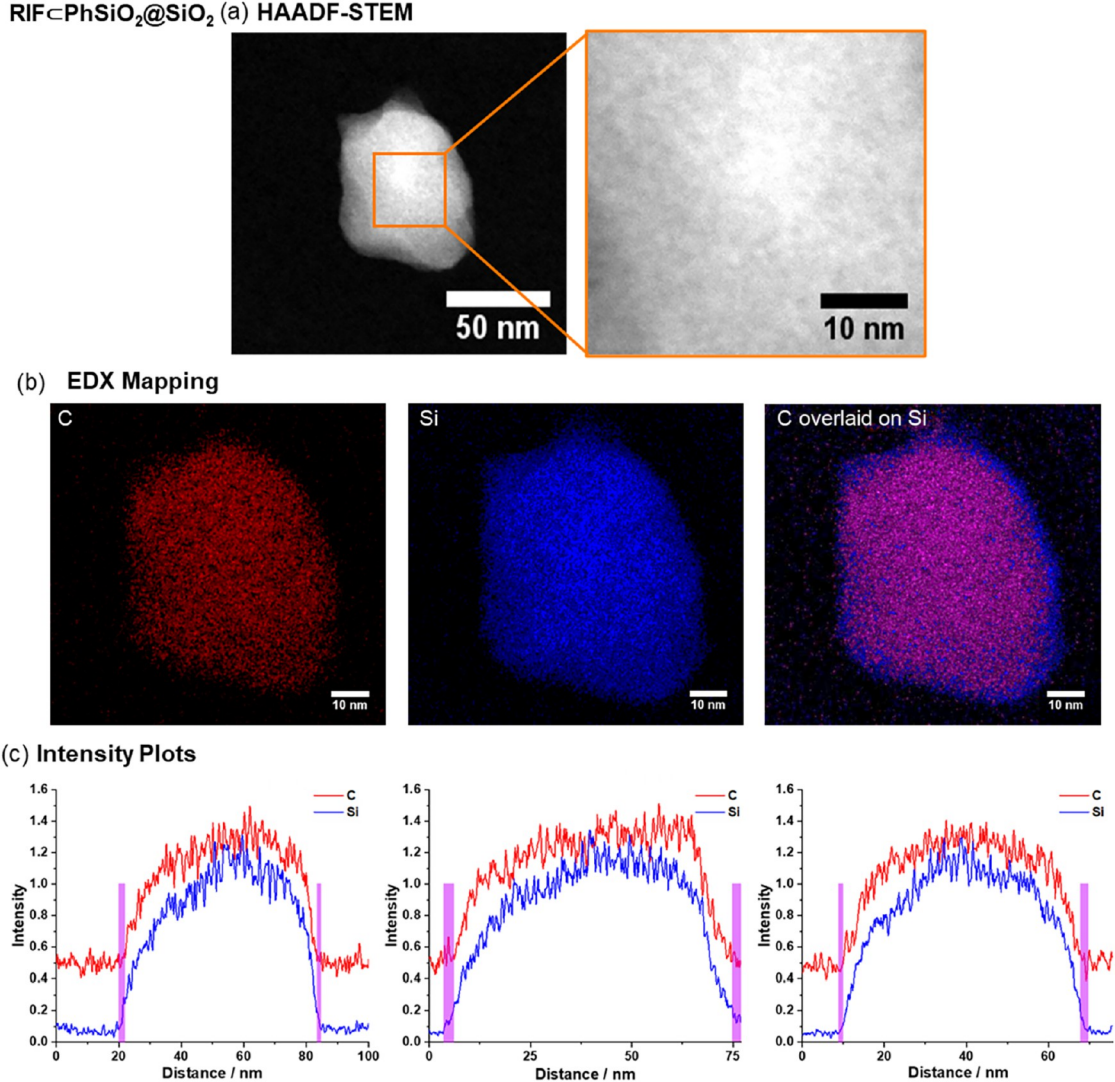
Electron microscopy analyses of **RIF⊂PhSiO**_**2**_ **@SiO**_**2**_: (a) HAADF-STEM; (b) EDX mapping of carbon (red), silicon (blue), and overlaid carbon–silicon distributions with overlapping areas in magenta; (c) intensity profiles of silicon and carbon signals across the nanoparticle plotted from a straight line drawn across the nanoparticle (a purple bar, 1–4 nm, highlights the differences between carbon and silicon distributions).

**Figure 5:**
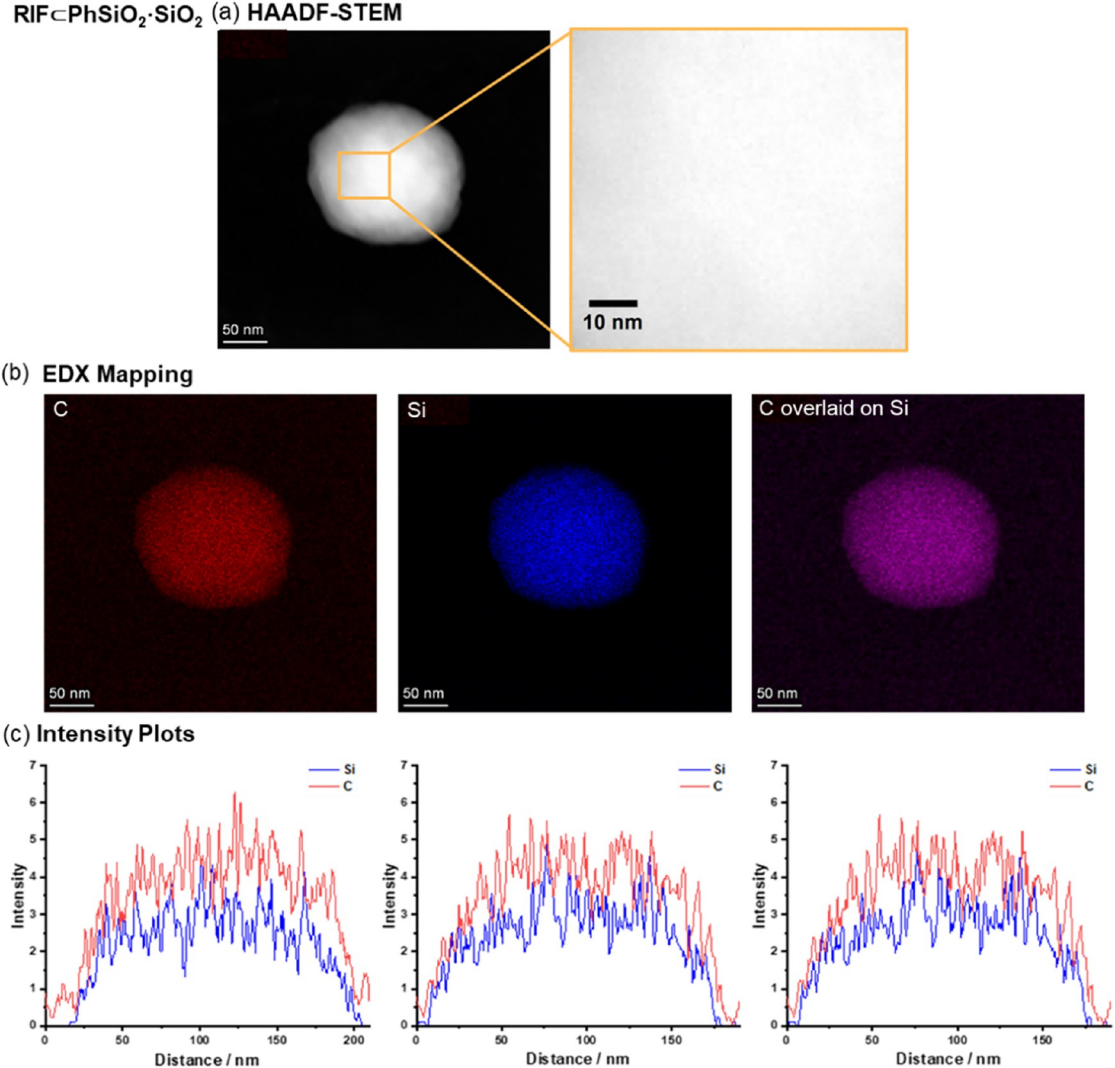
Electron microscopy analyses of **RIF⊂PhSiO**_**2**_ ***·*** **SiO**_**2**_: (a) HAADF-STEM; (b) EDX mapping of carbon (red), silicon (blue), and overlay of carbon–silicon distributions; and (c) intensity profiles of silicon and carbon signals across the nanoparticle plotted from a straight line drawn across the nanoparticle.

To probe structural and compositional differences in nanoparticles synthesized via the core@shell and co-condensation methods, we employed quantitative ^29^Si solid-state magic-angle spinning nuclear magnetic resonance (MAS NMR) spectroscopy and compared the two sets of nanoparticles, **RIF⊂PhSiO**_**2**_ **@SiO**_**2**_ and **RIF⊂PhSiO**_**2**_ **·SiO**_**2**_, with corresponding particles without rifampicin, **PhSiO**_**2**_ **@SiO**_**2**_ and **PhSiO**_**2**_ **·SiO**_**2**_, as well as plain SiO_2_ (Figure 6a). ^29^Si signals corresponding to four characteristic Si sites (Q^4^, Q^3^, T^3^, and T^2^ sites)^49^ in the organosiloxane framework of the particle are observed (Figure 6b), with minor intensity between Q^3^ and T^3^ signals suggesting the presence of trace Q^2^ sites.

**Figure 6:**
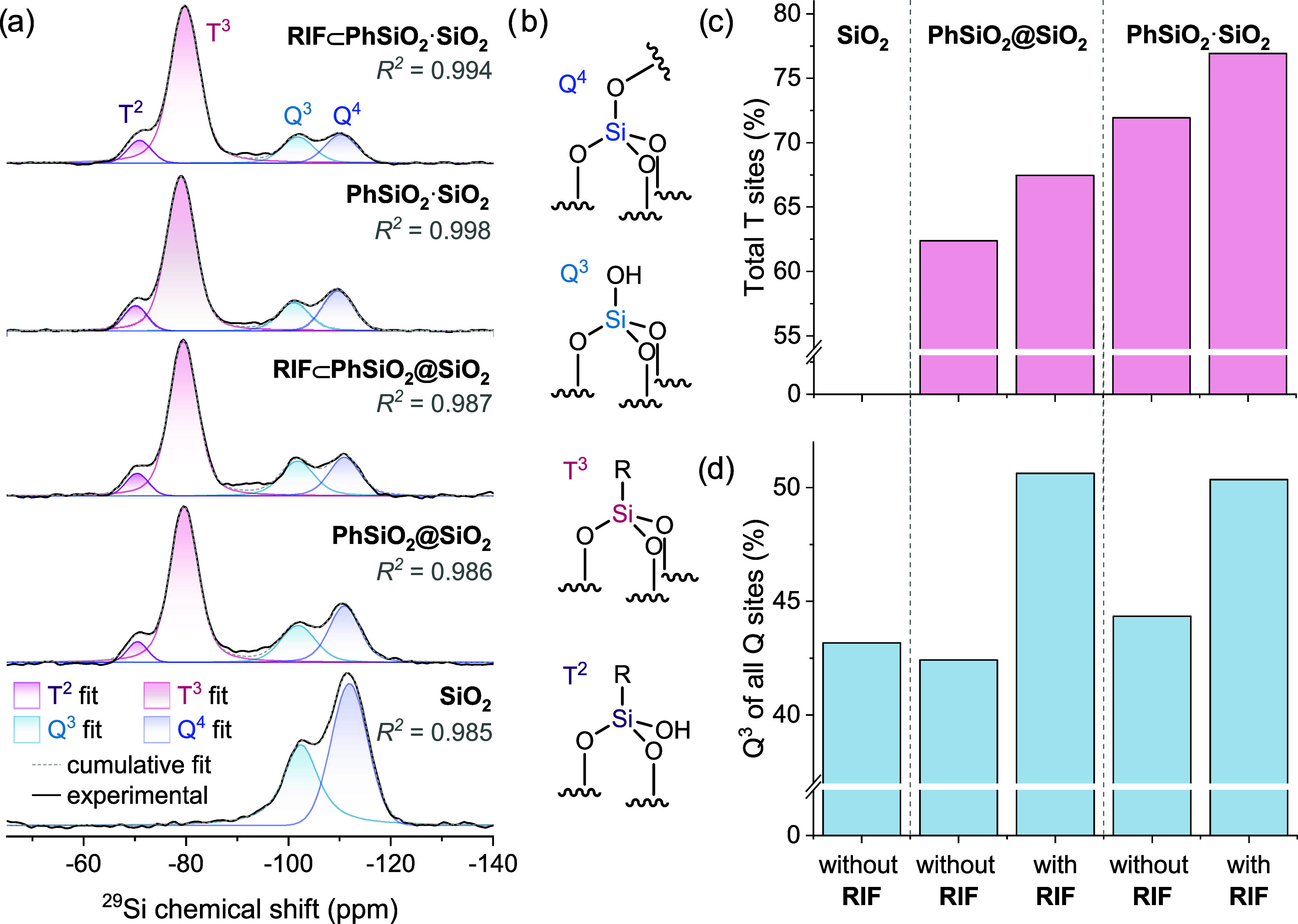
Structural characterization of silica nanoparticles by solid-state magic-angle spinning nuclear magnetic resonance (MAS NMR) spectroscopy. (a) Quantitative ^29^Si MAS NMR (14.1 T, 8 kHz) of **PhSiO**_**2**_ **@SiO**_**2**_ and **PhSiO**_**2**_ **·SiO**_**2**_ silica nanoparticles, with and without encapsulated rifampicin; (b) structures of observed Si sites in nanoparticles. (c, d) Compositional analysis of nanoparticles extracted by fitting ^29^Si NMR spectra showing variation in the total proportion of T sites (T^3^ + T^2^) to Q sites in each material (c) and the proportion of Q^3^ sites from all Q sites (Q^4^ + Q^3^) (d).

A marked increase in the ratio of total T sites (T*^n^*) to total Q sites (Q*^n^*) is seen in **PhSiO**_**2**_ **·SiO**_**2**_ versus **PhSiO**_**2**_ **@SiO**_**2**_ particles (Figure 6c). Synthetically, T*^n^* sites originate from the condensation of the organosilane precursor to give [SiO*_n_* (OH)_3‑*n*_R] (1 ≤ *n* ≤ 3) environments in the silica network and, in **PhSiO**_**2**_ **@SiO**_**2**_, are concentrated in the particle core. Thus, the increased number of T*^n^* sites observed in co-condensed **PhSiO**_**2**_ **·SiO**_**2**_ particles confirms that the silsesquioxane network extends throughout a greater proportion than in core@shell particles, at the expense of siloxane [SiO*_n_* (OH)_4‑*n*_] (1 ≤ *n* ≤ 4) Q*^n^* sites. These studies support the electron microscopy results and analysis by EDX mapping for the difference in the distribution of the organosilane framework, which is throughout the co-condensed particles in contrast to the core only for **PhSiO**_**2**_ **@SiO**_**2**_ particles.

Inclusion of hydrophobic rifampicin during particle synthesis via either method also increases the ratio of T*^n^* to Q*^n^* sites (Figure 6c), suggesting that the presence of rifampicin promotes the formation of a more extensive silsesquioxane network. For **RIF⊂PhSiO**_**2**_ **@SiO**_**2**_, this is expected to occur only during the formation of the core, as rifampicin is introduced at this stage in the synthesis and encapsulated in the core. This higher density of organosilane species is consistent with the formation of cavities featuring phenylsilane-terminated interior surfaces suitable for the encapsulation of hydrophobic rifampicin. Interestingly, however, rifampicin encapsulation also significantly alters Q-site connectivity, with the proportion of reduced-connectivity Q^3^ sites increasing at the expense of Q^4^ sites (Figure 6d). This suggests that rifampicin may interact with the interior surfaces of the particle not only via its hydrophobic moieties, but also by interaction between its hydrophilic functional groups and the Si–OH functionality present in Q^3^ but not Q^4^ sites.

Overall, ^29^Si NMR confirms that significant structural changes result from altering the synthetic route to organosilane nanoparticles, consistent with the expected homogeneous network and core@shell structural models. Furthermore, rifampicin encapsulation also induces detectable structural changes that suggest that rifampicin populates cavities within the nanoparticles, interacting with interior particle surfaces via both hydrophobic and hydrophilic moieties.

To evaluate the release of rifampicin *in vitro* under static and ultrasound conditions, quantification by liquid chromatography–mass spectrometry (LC-MS) was performed (Figure S8). Under static conditions in water, neither **RIF⊂PhSiO**_**2**_ **@SiO**_**2**_ nor **RIF⊂PhSiO**_**2**_ **·SiO**_**2**_ released a detectable amount of rifampicin, as expected for the encapsulated antibiotic and in contrast with mesoporous silica nanoparticles.^28^ LFUS was applied for 10 s (29 kHz, 0.27 W) to 2 mg/mL aqueous nanoparticle suspensions using ultrasound conditions optimized for the treatment of biological samples. Under these conditions, rifampicin release from core@shell nanoparticles **RIF⊂PhSiO**_**2**_ **@SiO**_**2**_ equated to 4.6 ± 0.1 ng_**RIF**_/mg_NP_. However, drug release from co-condensed particles **RIF⊂PhSiO**_**2**_ **·SiO**_**2**_ was below the limit of detection. The morphology of the **RIF⊂PhSiO**_**2**_ **@SiO**_**2**_ nanoparticles post-sonication was examined by TEM, but no significant differences were observed in morphology or size. Interestingly, quantification of rifampicin release from **RIF+NR⊂PhSiO**_**2**_ **@SiO**_**2**_ yielded 4.6 ± 0.1 ng_RIF_/mg_NP_, comparable to that of **RIF⊂PhSiO**_**2**_ **@SiO**_**2**_, indicating that the presence of Nile red did not affect the release under 10s LFUS application.

To further confirm the ultrasound effect, we examined the release of rifampicin from **RIF⊂PhSiO**_**2**_ **@SiO**_**2**_ versus the duration of LFUS application using the ultrasound conditions described above. Increased rifampicin release was observed, reaching a plateau at 7.8 ± 0.8 ng_RIF_/mg_NP_ after sonication for 60 s (Figure S8c). These results support our previous findings for the release of the encapsulated agent based on the mechanical effects of cavitation in solution. The formation of bubbles during cavitation of the particles by LFUS was previously shown to be particularly effective for amorphous, nonporous silica nanoparticles, which encapsulate the agent, rather than the mesoporous designs, which rely on adsorption of the agent in the porous structure.^26,28^ For the latter, there is no effect of LFUS at different powers or times, and the release is uncontrolled, similar to static release. Temperature change is also not a dominating factor in the release. Minimal change (0.17 ± 0.13 °C) in overall temperature was observed during sonication; however, localized temperature increases may occur.^50,51^ The **RIF⊂PhSiO**_**2**_ **@SiO**_**2**_ nanoparticles were selected for further studies over **RIF⊂PhSiO**_**2**_ **·SiO**_**2**_ due to the lack of release observed for the latter at the ultrasound conditions for biofilm experiments.

### 2.2 Bacterial Studies of the RIF⊂PhSiO_2_@SiO_2_ upon LFUS against *S. aureus*

To investigate the bacterial biofilm killing effects of **RIF⊂PhSiO**_**2**_ **@SiO**_**2**_, **RIF+NR⊂PhSiO**_**2**_ **@SiO**_**2**_, and **PhSiO**_**2**_ **@SiO**_**2**_ with the application of ultrasound, single-species *S. aureus* (strain SH1000) planktonic cultures and 72 h biofilms were studied. The minimum nanoparticle concentration for inhibition (MNCI) of **PhSiO**_**2**_ **@SiO**_**2**_, **RIF⊂PhSiO**_**2**_ **@SiO**_**2**_, and **RIF+NR⊂PhSiO**_**2**_ **@SiO**_**2**_ before and after ultrasound application (10 s) was assessed against planktonic cultures of *S. aureus* (Figure S9). These values were determined as the minimum particle concentration at which no bacterial growth was observed. Without exposure to ultrasound, the MNCI of **RIF⊂PhSiO**_**2**_ **@SiO**_**2**_ and **RIF+NR⊂PhSiO**_**2**_ **@SiO**_**2**_ were determined to be 62 μg/mL and 16 μg/mL, respectively. After ultrasound application (10 s), the MNCI values decreased to 32 μg/mL for **RIF⊂PhSiO**_**2**_ **@SiO**_**2**_ and 8 μg/mL for **RIF+NR⊂PhSiO**_**2**_ **@SiO**_**2**_. Both sets of rifampicin-containing nanoparticles exhibited a 2-fold reduction in MNCI on application of ultrasound, suggesting that an increased amount of rifampicin was released with ultrasound application. **PhSiO**_**2**_ **@SiO**_**2**_ did not exhibit antimicrobial activity up to 10 mg/mL with and without ultrasound, confirming that the nanoparticles without rifampicin were not bactericidal. Moreover, core@shell nanoparticles showed low overall cytotoxicity against H400 epithelial cells and macrophages, as assessed by MTT assays (Figure S10).

The triggered release and delivery of rifampicin to biofilms with **RIF⊂PhSiO**_**2**_ **@SiO**_**2**_ was investigated using a mature 72 h *S. aureus* biofilm.^26^ The effect of the different treatments on the biofilm and the bacterial morphology was explored by Scanning Electron Microscopy (SEM). *S. aureus* typically displays clusters of round-shaped cocci, which were not affected by the application of ultrasound (Figure 7). We examined the bacterial cell morphology upon treatment with plain core@shell nanoparticles, **PhSiO**_**2**_ **@SiO**_**2**_, with and without ultrasound (Figure 7d–f). In static conditions, large nanoparticle clusters were observed covering the majority of the biofilm surface (Figure 7d). The application of ultrasound reduces the amount of clustering, which enhances penetration (Figure 7e,f). Minimal morphological changes in the *S. aureus* biofilms were observed when **RIF⊂PhSiO**_**2**_ **@SiO**_**2**_ was added without sonication; however, in combination with ultrasound, extensive membrane damage and debris were observed, suggesting that **RIF⊂PhSiO**_**2**_ **@SiO**_**2**_, in combination with ultrasound, disrupts the structure of the biofilms and leads to enhanced bacterial eradication within *S. aureus* biofilms (Figures 7g–i and S11).

**Figure 7:**
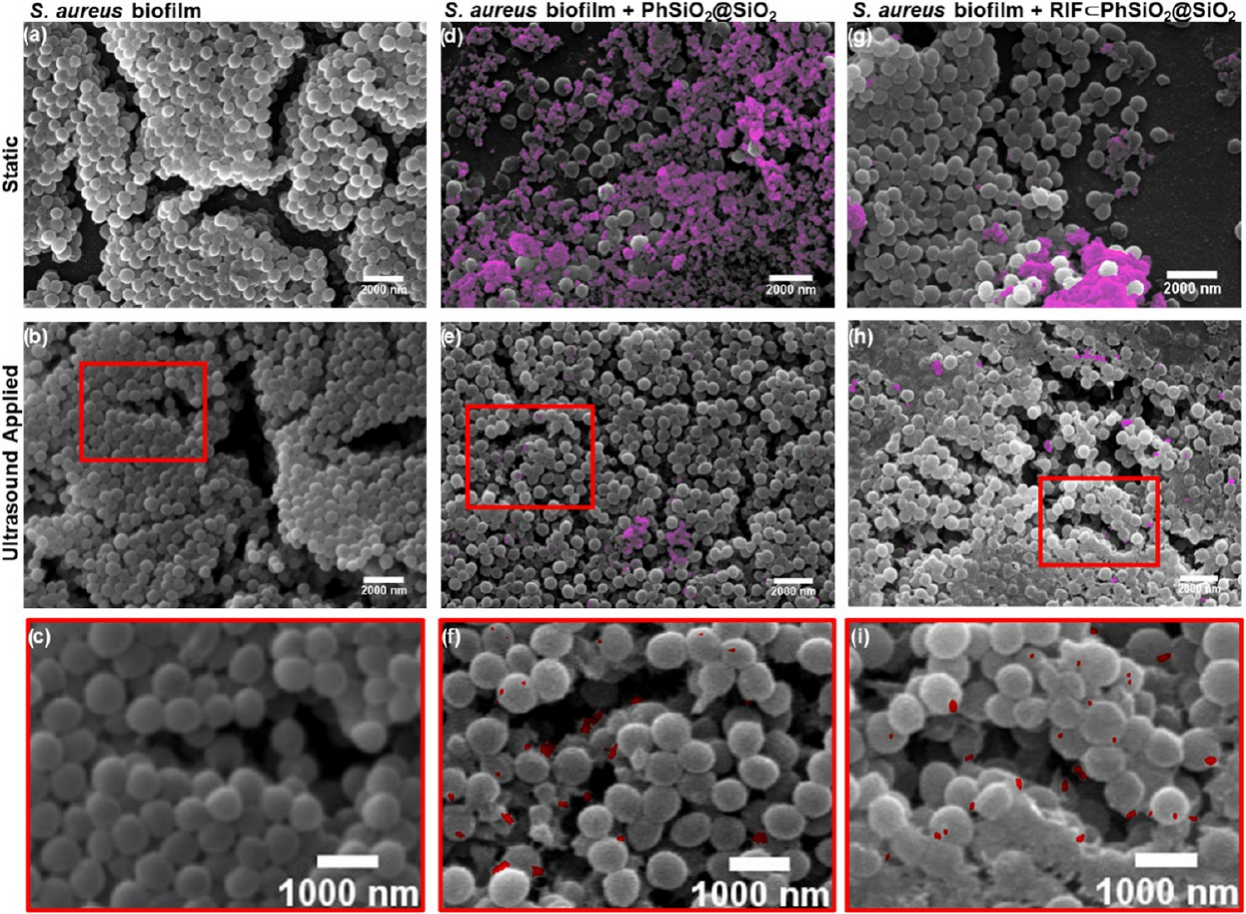
SEM images showing the effect of treatment with nanoparticles before and after ultrasound (0.27 W, 10 s) in *S. aureus* 72 h biofilms showing static conditions (a, d, g) and post-ultrasound (b, e, h) for treatment with particle with no rifampicin, **PhSiO**_**2**_ **@SiO**_**2**_ (d, e), and particles with rifampicin, **RIF⊂PhSiO**_**2**_ **@SiO**_**2**_ (g, h). Insets shown for each sample treated with ultrasound (c, f, i). The accumulation of large nanoparticle clusters is highlighted in magenta, with smaller clusters in red.

The effects of combined treatment of *S. aureus* biofilms with rifampicin-encapsulated nanoparticles, **RIF⊂PhSiO**_**2**_ **@SiO**_**2**_, **RIF+NR⊂PhSiO**_**2**_ **@SiO**_**2**_, and LFUS were investigated further by colony counting and biofilm viability assays in parallel as well as confocal laser scanning microscopy of biofilms stained with dyes Syto 9 and propidium iodide and subsequent analysis using the Biofilm Viability Checker to calculate the percentage of live and dead cells (Figure 8).^30^ Syto 9 enters all bacteria, whereas propidium iodide can only enter bacteria with a compromised envelope, thereby acting as a marker of dead cells. The *S. aureus* biofilms were treated for the effect of a short ultrasound application (10 s) together with the rifampicin-encapsulated nanoparticles **RIF⊂PhSiO**_**2**_ **@SiO**_**2**_ and **RIF+NR⊂PhSiO**_**2**_ **@SiO**_**2**_; the studies were compared to the plain drug, as well as control particles without rifampicin, **PhSiO**_**2**_ **@SiO**_**2**_ and **NR⊂PhSiO**_**2**_ **@SiO**_**2**_. Stained biofilms show a small decrease in cell viability for plain rifampicin, and application of ultrasound had no significant effect (Figure 8a,b). This is expected due to the hydrophobic nature of the antibiotic; even with LFUS application, the penetration is not significantly enhanced. The application of rifampicin-encapsulated particles, **RIF⊂PhSiO**_**2**_ **@SiO**_**2**_, shows a strong reduction of cell viability on application of ultrasound from 80 to 10%. Independent CFU counting supports the results with the rifampicin-containing nanoparticles showing the biggest change upon ultrasound treatment: 800-fold reduction from log_10_(CFU/mL) 11.0 ± 1.0 to 8.1 ± 1.3 (p = 0.02) for **RIF⊂PhSiO**_**2**_ **@SiO**_**2**_ and a 400-fold reduction for **RIF+NR⊂PhSiO**_**2**_ **@SiO**_**2**_ from log_10_(CFU/mL) 10 ± 1 pre-ultrasound to 7.4 ± 0.2 post-ultrasound (Figure 8c). These results showed that the combination of **RIF⊂PhSiO**_**2**_ **@SiO**_**2**_ or **RIF+NR⊂PhSiO**_**2**_ **@SiO**_**2**_ with ultrasound remarkably improves the performance of rifampicin in the treatment of *S. aureus* biofilms. A colorimetric biofilm viability assay using MTT (Figure 8d) further supports the aforementioned biofilm assay results. The biofilm viability assay determined a significant difference (*p* < 0.1) between the biofilm viability for static and sonicated **RIF⊂PhSiO**_**2**_ **@SiO**_**2**_ (1 mg/mL) samples, where bacterial cell death was greater on application of ultrasound, decreasing from an OD_570_ value of 0.50 to 0.22. Results for the sonicated **RIF⊂PhSiO**_**2**_ **@SiO**_**2**_ particles differed significantly (*p* < 0.1) from treatment with free rifampicin with ultrasound (OD_570_ = 0.22 vs OD_570_ = 0.37), where greater bacterial cell death was observed for the nanoparticles than the free drug, confirming that the synergistic effects of ultrasound and **RIF⊂PhSiO**_**2**_ **@SiO**_**2**_ are an essential combination for biofilm eradication.

**Figure 8:**
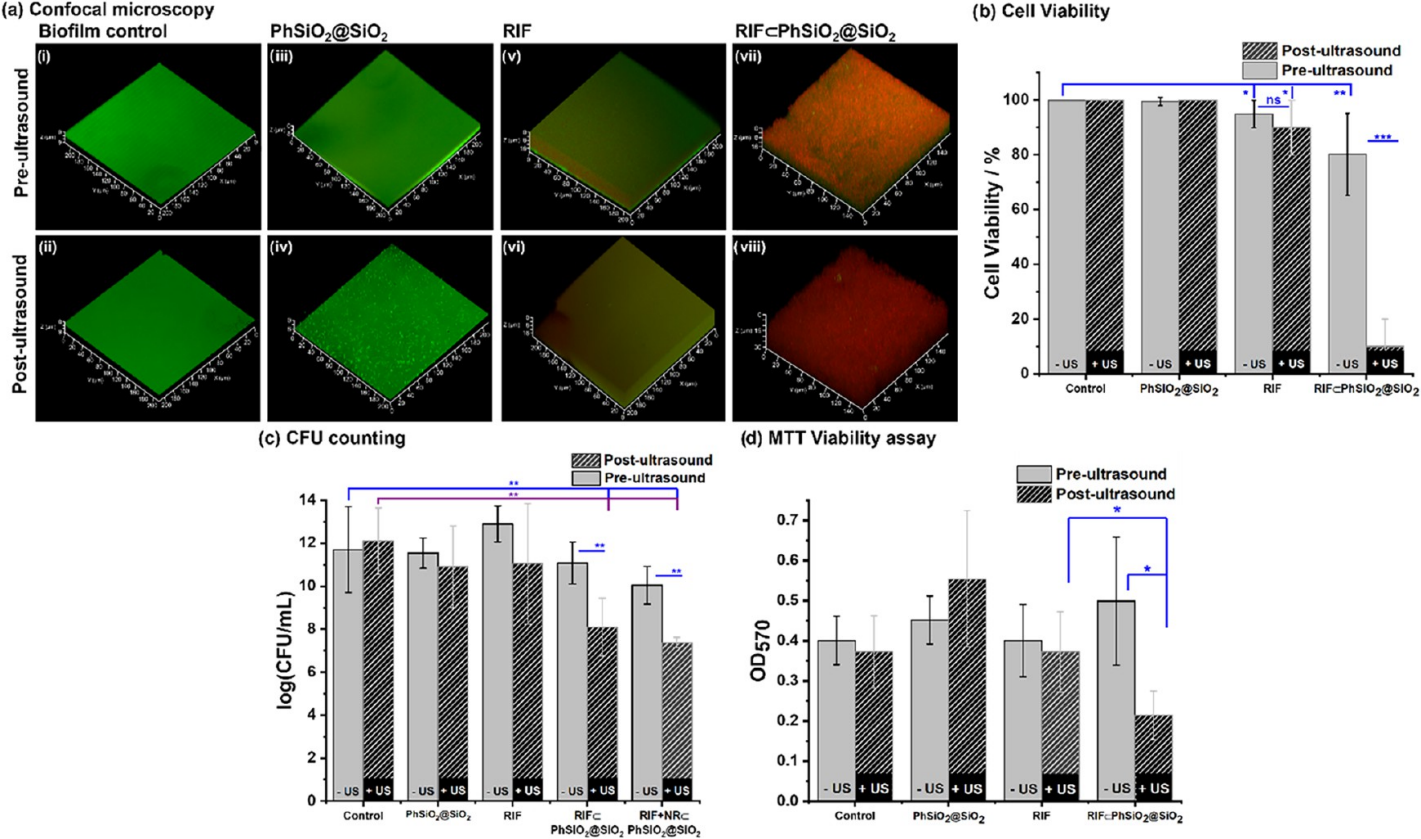
(a) 3D confocal laser scanning microscopy fluorescence images showing the effect of treatment with nanoparticles or rifampicin with and without the application of ultrasound against *S. aureus* biofilms, using dye staining (*n* = 3). (b) Quantification of bacterial viability in biofilm using the biofilm staining imaging analysis. (c) Independent assay for CFU counting and (d) MTT viability assay for *S. aureus* biofilm cell viability (n=3). Mean and standard deviation of biofilms tested for statistical significance using a two-tailed *t* test (*p < 0.5, **p < 0.05, ***p < 0.005).

### 2.3 Nanoparticle Penetration in Biofilms

To correlate the biofilm eradication to nanoparticle penetration, we analyzed in independent confocal fluorescence microscopy experiments the bacterial viability and the nanoparticle penetration across the layers of the biofilm treated with rifampicin-encapsulated nanoparticles. Biofilms treated with **RIF⊂PhSiO**_**2**_ **@SiO**_**2**_ without ultrasound indicated that in all cases bacterial cell death was primarily observed in the top layers of the biofilms, and only upon the application of ultrasound did the penetration of the nanoparticles show the highest eradication across the entire biofilm (Figures 9 and S13). These results are indicative of the crucial synergistic capabilities of **RIF⊂PhSiO**_**2**_ **@SiO**_**2**_ and ultrasound, suggesting that the application of ultrasound not only enables the release of the drug from the nanoparticles but also facilitates the penetration of the nanoparticles deeper into the biofilm.

**Figure 9:**
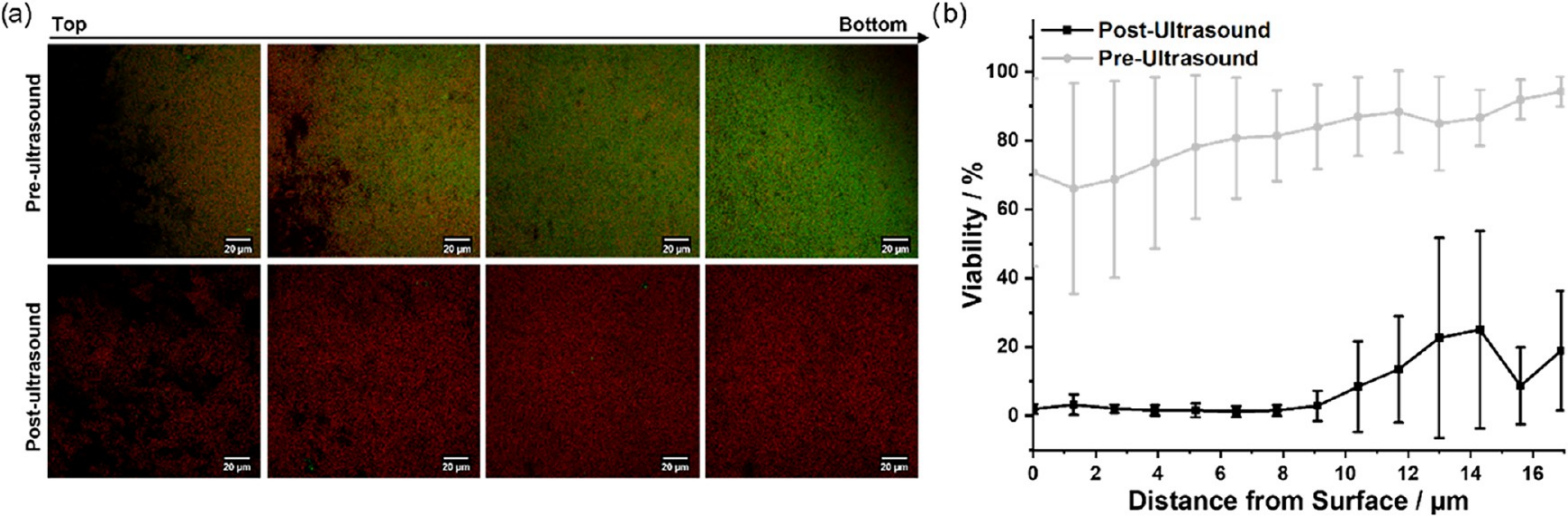
(a) Selected confocal fluorescence images (from the 3D z-stack) showing the effects of treatment with **RIF⊂PhSiO**_**2**_ **@SiO**_**2**_ (1 mg/mL) across S. aureus biofilms, without and with the application of ultrasound (0.27 W, 10 s). Quantification of biofilm viability using dye staining (*n* = 3). (b) Plot of viability against distance from the surface of biofilms from processing of individual image slices using an automated image analysis package within ImageJ (Biofilm Viability Checker).^30^

To visualize the nanoparticle presence throughout the biofilm layers, we employed the fluorescent nanoparticles, **RIF+NR⊂PhSiO**_**2**_ **@SiO**_**2**_, where Nile red is coencapsulated with the antibiotic and emits the characteristic Nile red fluorescence signal. It is important to note that Nile red is not released upon LFUS application, as shown in the aforementioned, independent nanoparticle experiments. Confocal fluorescence microscopy z-stack images of the biofilm were analyzed, visualizing the nanoparticles with the red fluorescent signal, **RIF+NR⊂PhSiO**_**2**_ **@SiO**_**2**_, and the biofilm layers with green Syto 9 stain. Biofilms treated with nanoparticles and no ultrasound (Figure 10a) show nanoparticles mostly in the top layers of the biofilm, as shown by the purple arrow, in an estimated depth of 1.6 μm of the overall 5.9 μm thick biofilm. In this case, only diffusion allowed the nanoparticle presence in the upper layers of the biofilm. Upon the application of ultrasound, the analysis of 3D confocal imaging shows particles located far deeper into the bottom of the biofilm (purple arrow), estimated to be 5.6 μm. An increased concentration of particles is observed in the biofilms treated with ultrasound, possibly due to the immediate penetration of the particles, whereas in the pre-ultrasound conditions, particles are washed away. These data show that the application of LFUS has a strong impact in nanoparticle delivery and penetration within biofilms, in agreement with the dye-staining assays showing enhanced drug delivery.

**Figure 10:**
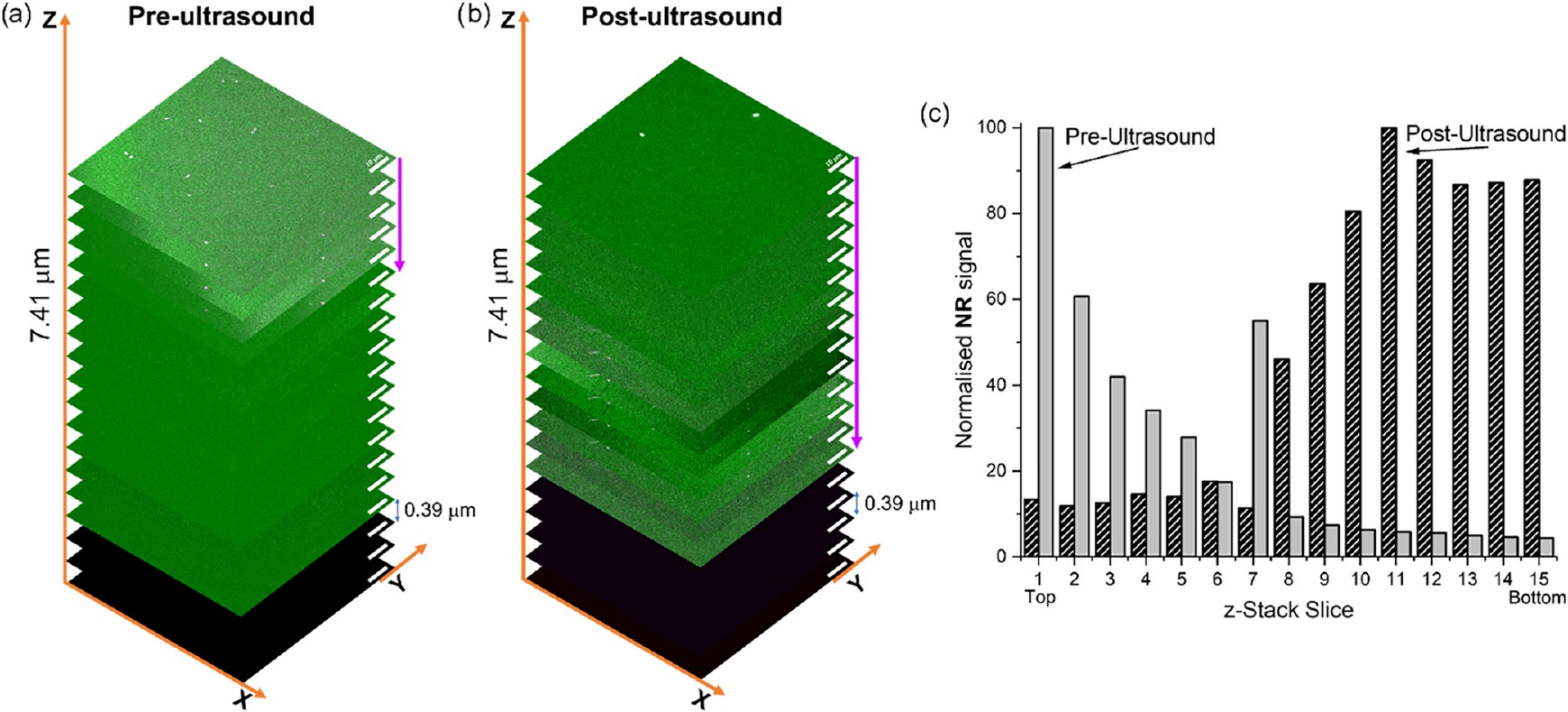
Confocal fluorescence images of Syto 9-stained 72-h *S. aureus* biofilms treated with **RIF+NR⊂PhSiO**_**2**_ **@SiO**_**2**_ (1 mg/mL) (a) before ultrasound application and (b) post-ultrasound application in a z-stack. Scale bars represent 10 μm in the *x* and *y* axes (*n* = 2). Purple arrows indicate the penetration of the **RIF+NR⊂PhSiO**_**2**_ **@SiO**_**2**_ nanoparticles. (c) Quantification of Nile red luminescence detected at each depth through *S. aureus* biofilm.

## Conclusions

We have shown that the encapsulation of hydrophobic drugs during silica nanoparticle preparation is an effective design strategy for the temporal control of their delivery via ultrasound. Selective encapsulation of rifampicin within the hydrophobic core of core@shell nanoparticles, followed by coating of a hydrophilic shell, provides particles showing the most effective drug delivery upon the application of ultrasound. Solid-state NMR shows that the incorporation of rifampicin within the core of such particles leads to a higher density of organosilane species ([SiO*_n_* (OH)_3‑*n*_R]), consistent with drug encapsulation with hydrophobic cavities inside the core. Both spectroscopic and electron microscopy techniques provide information on the core@shell vs the co-condensed silica nanoparticles to support the encapsulation and release studies. The amorphous, nonporous, and hydrophilic shell provides compatibility for biofilm penetration and allows drug release to be activated only upon low-frequency ultrasound based on cavitation effects. The rifampicin-encapsulated nanoparticles show effective antibacterial activity against *S. aureus* biofilms only triggered upon a short 10 s application of LFUS, as measured by bacterial growth assays and biofilm viability assays, and lead to 90% biofilm eradication. They do not show toxicity against H400 human epithelial cells, highlighting their applicability and potential for translation. Nanoparticle penetration through the biofilm is evident from SEM imaging and confocal fluorescence microscopy. Ultrasound application alone does not lead to the same penetration of the free drug. A unique advantage of the core@shell silica design is the coencapsulation of fluorescent probes such as Nile red along with rifampicin. This allows the tracking of the fluorescent nanoparticles through the biofilm during the drug, independent of the bacterial staining assays. Tracking the Nile red signal of the fluorescent nanoparticles by confocal microscopy shows the presence of the particles at the bottom of biofilms upon ultrasound application, supporting the efficient eradication of biofilms observed by biofilm assays. This work introduces and demonstrates nonporous, amorphous silica nanoparticle design as a powerful approach for deep penetration delivery of hydrophobic drugs with spatial and temporal control triggered by LFUS, opening a path to localized delivery and therapy for a wider range of drugs. Moreover, we anticipate that this nanoparticle design strategy will allow the repurposing of antibiotics and other antimicrobials, which have been challenging to deliver, helping to decrease overall drug doses, and will also inspire the development of other similar drug delivery systems for the localized treatment of other pathologies, such as cancer.

## Supplementary Material



## Notes

Experimental details of materials preparation, methodology, and instrumentation, additional nanoparticle characterization, as well as additional bacterial studies are reported (PDF)

## ◆

Institute of Materials Science of Barcelona (ICMAB-CSIC), Campus of the UAB, 08193 Bellaterra, Spain
