# Supplementary material for: Ultrasound-Responsive Nanoparticles Enable Hydrophobic Antibiotic Release and Deep Penetration for Biofilm Treatment
Source: JACS Au. 2026 Feb 25;6(3):1847–59. doi: 10.1021/jacsau.5c01711 (PMC13014244; doi:10.1021/jacsau.5c01711)
Supplement: Supplementary file 1 [file au5c01711_si_001.pdf]

Supporting Information

Ultrasound-Responsive Nanoparticles Enable Hydrophobic Antibiotic Release and Deep Penetration for Biofilm Treatment

Maria L. Odyniec,[a†] Daniel J. Bell,[a†] Benjamin M. Gallant,[a] Rininta Firdaus,[a,b] Grace Ball,[a] Liam Hughes,[c] Rebecca Oxtoby,[d] Benjamin J. Hewitt,[b,e] Christopher M. Williams,[a] Asier R. Muguruza,[a] Tim W. Overton,[f] Hung-Ji Tsai,[d] Yu-Lung Chiu,[c] Dominik J. Kubicki,[a] A. Damien Walmsley,[b] Sarah A. Kuehne, [b,g*] Zoe Pikramenou[a*]

a. *School of Chemistry, College of Engineering and Physical Sciences, University of Birmingham, Edgbaston, Birmingham B15 2TT, UK. E-mail: z.pikramenou@bham.ac.uk*
b. *School of Dentistry, College of Medical and Dental Sciences, University of Birmingham, Edgbaston, Birmingham B15 2TT, UK.*
c. *School of Metallurgy and Materials, College of Engineering and Physical Sciences, University of Birmingham, Edgbaston, Birmingham, B15 2TT, UK.*
d. *School of Biosciences, Institute of Microbiology and Infection, University of Birmingham, Edgbaston, Birmingham, B15 2TT, UK.*
e. *School of Biomedical Sciences, College of Medicine and Health, University of Birmingham, Edgbaston, Birmingham B15 2TT, UK*
f. *School of Chemical Engineering, College of Engineering and Physical Sciences, University of Birmingham, Edgbaston, Birmingham, B15 2TT, UK.*
g. *School of Science and Technology, Nottingham Trent University, Nottingham, UK*

Table of contents

Nanoparticle Synthesis S2

Methods and Instrumentation S3

Biological Studies S5

Additional Nanoparticle Characterisation S8

Further Biological Studies S14

Additional References S17

Nanoparticle Synthesis

Materials. Phenyltrimethoxysilane (PhTMS) and rifampicin (RIF) were supplied by Sigma Aldrich; tetraethyl orthosilicate (TEOS) and ethanol was supplied by Fisher Scientific; and ammonium hydroxide solution (28-30 %) and Nile Red (NR) were supplied by Thermo Scientific. All starting materials were used without purification. MTT was supplied by Thermo Fisher. Propidium iodide and Syto9 were supplied in a Biofilm Viability Kit from Thermo Fisher.

Synthesis

General procedure 1 for core@shell particles. **$PhSiO_2@SiO_2$.** A solution of milliQ water (3.0 mL, 166.5 mmol, 4.76 M), 28-30% w/w ammonium hydroxide solution (7.0 mL, 103.6 mmol, 2.96 M) and ethanol (25 mL) was prepared. Step 1: Preparation of core $PhSiO_2$ PhTMS (1.17 g, 1.1 mL, 5.89 mmol, 0.168 M) was added, and stirred for 1 h at room temperature. Particles were examined by TEM and DLS from the reaction mixture *via* a 1:100 dilution in water. Step 2: TEOS (0.56 g, 0.6 mL, 2.69 mmol, 0.077 M) was added and stirred for 16 h. $PhSiO_2@SiO_2$ nanoparticles were isolated by centrifugation (15 min, 7830 rpm) and washed with ethanol (20 mL) and water (2 x 20 mL). After three cycles of the centrifugation-washing procedures, the nanoparticles were dried in-vacuo to yield a white powder (0.62 g). Diameter = 50 ± 25 nm (DLS_{number}), PDI = 0.25; 59 ± 19 nm (TEM), ζ-potential = -32 ± 6 mV, FT-IR = 3073 (C-H, stretch), 1594 (C-C, stretch), 1430 (C-C, stretch), 1087 (Si-O-Si, stretch), 1028 (Si-C, bend), 998 (Si-C), 780 (Si-O, symmetric stretch), 728 (Si-C, stretch), 695 (Si-O, symmetric bend), 482 (Si-O-Si, asymmetric bend) cm^{-1}.

$RIF \subset PhSiO_2@SiO_2$. Following general procedure 1, rifampicin (40.0 mg, 0.049 mmol, 1.40 mM) was added to the initial reaction solution, before PhTMS addition. Nanoparticles were isolated as a pale orange powder (0.70 g). Diameter = 90 ± 20 nm (DLS_{number}), PDI = 0.26; 92 ± 25 nm (TEM), ζ-potential = -46 ± 7 mV, FT-IR = 3074 (C-H, stretch), 1595 (C-C, stretch), 1430 (C-C, stretch), 1079 (Si-O-Si, asymmetric stretch), 1027 (Si-C, bend), 998 (Si-OH, stretching), 781 (Si-O, symmetric stretch), 728 (Si-C, stretch), 694 (Si-O, symmetric bend), 482 (Si-O-Si, asymmetric bend) cm^{-1}.

$NR \subset PhSiO_2@SiO_2$. Following general procedure 1, Nile red (0.1 mg, 0.314 μmol, 8.98 μM) was added to the initial reaction solution, before PhTMS addition. Nanoparticles were isolated as a pale pink powder (0.68 g). Diameter = 90 ± 18 nm (DLS_{number}), PDI = 0.39; 51 ± 17 nm (TEM), ζ-potential = -28 ± 8 mV, FT-IR = 3074 (C-H, stretch), 1595 (C-C, stretch), 1430 (C-C, stretch), 1081 (Si-O-Si, asymmetric stretch), 1028 (Si-C, bend), 998 (Si-OH, stretching), 782 (Si-O, symmetric stretch), 728 (Si-C, stretch), 695 (Si-O, symmetric bend), 482 (Si-O-Si, asymmetric bend) cm^{-1}.

$RIF+NR \subset PhSiO_2@SiO_2$. Following general procedure 1, rifampicin (40.0 mg, 0.049 mmol, 1.40 mM) and Nile red (0.1 mg, 0.314 μmol, 8.98 μM) were added to the initial reaction solution, before PhTMS addition. Nanoparticles were isolated as a pale pink powder (0.72 g). Diameter = 160 ± 47 nm (DLS_{number}), PDI = 0.27; 87 ± 13 nm (TEM), ζ-potential = -22 ± 9 mV, FT-IR = 3072 (C-H, stretch), 1596 (C-C, stretch), 1432 (C-C, stretch), 1088 (Si-O-Si, asymmetric stretch), 1028 (Si-C, bend), 996 (Si-OH, stretching), 782 (Si-O, symmetric stretch), 728 (Si-C, stretch), 696 (Si-O, symmetric bend), 484 (Si-O-Si, asymmetric bend) cm^{-1}.

General procedure 2 for particles from co-condensation **$PhSiO_2 \cdot SiO_2$.** A solution of milliQ water (3.0 mL, 166.5 mmol, 4.76M), 28-30% w/w ammonium hydroxide solution (7.0 mL, 103.6 mmol, 2.96 M) and ethanol (25 mL) was prepared. PhTMS (1.17 g, 1.1 mL, 5.89 mmol, 0.168 M) and TEOS (0.56 g, 0.6 mL, 2.69 mmol, 0.077 M) were added simultaneously, and the reaction mixture was stirred for 17 h. $PhSiO_2 \cdot SiO_2$ nanoparticles were isolated by centrifugation (15 min, 7830 rpm) and washed with ethanol (20 mL) and water (2 x 20 mL). After three cycles of the centrifugation-washing procedures, the nanoparticles were dried in-vacuo to yield a white powder (0.22 g). Diameter = 270 ± 57 nm (DLS_{number}), PDI = 0.39; 150 ± 15 nm (TEM), ζ-potential = -30 ± 10 mV, FT-IR = 3074 (C-H, stretch), 1595 (C-C, stretch), 1430 (C-C, stretch), 1047 (Si-O-Si, asymmetric stretch), 1028 (Si-C, bend), 998 (Si-OH, stretching), 781 (Si-O, symmetric stretch), 733 (Si-C, stretch), 695 (Si-O, symmetric bend), 483 (Si-O-Si, asymmetric bend) cm^{-1}.

$RIF \subset PhSiO_2 \cdot SiO_2$. Following general procedure 2, rifampicin (40.0 mg, 0.049 mmol, 1.40 mM) was added to the initial reaction solution, before the addition of PhTMS and TEOS. Nanoparticles were isolated as a pale orange powder (0.34 g). Diameter = 430 ± 60 nm (DLS_{number}), PDI = 0.69; 54 ± 6 nm (TEM), ζ-potential = -27 ± 5 mV, FT-IR = 3072 (C-H, stretch), 1596 (C-C, stretch), 1432 (C-C,

stretch), 1064 (Si-O-Si, asymmetric stretch), 1028 (Si-C, bend), 1000 (Si-OH, stretching), 780 (Si-O, symmetric stretch), 736 (Si-C, stretch), 696 (Si-O, symmetric bend), 484 (Si-O-Si, asymmetric bend) cm^{-1}.

Methods and Instrumentation

Dynamic light scattering (DLS)* and *ζ-potential data were recorded by a Malvern Panalytical Zetasizer ZS instrument equipped with a H-Ne 633 nm laser at 25 ºC and with a backscattering of 173° using milliQ water as a dispersant. The instrument was controlled with a Malvern DTS 7.03 software. All sizes were determined based on 5 measurements containing 11 runs on each. z-potential was determined after measuring 100 runs per sample at 140 V, 25 °C in triplicate.

UV-vis spectra of powders were recorded using a Cary 5000 dual beam UV-Vis spectrophotometer in reflectance mode (R%) equipped with a solid-state attachment. Spectra are reported using F(R)

$$F(R) = \frac{(1-R\%)^2}{(2R\%)} \quad \textit{(eq. 1)}$$

Scanning electron microscopy (SEM) images were recorded in a Zeiss EVO10 SEM under a high vacuum at 20 kV electron beam and 5,000-25,000x magnification. SEM samples were prepared by mounting onto a carbon coated aluminium stub before splutter coating with gold (Quorum Emitech K550X splutter coater).

Transmission electron microscopy (TEM) images were recorded on a JEOL 1400 TEM or a TALOS F200X STEM. TEM samples were prepared by placing 0.1 mg/mL of silica particles in 1 mL of MilliQ water, then a drop of the suspension was placed on a TEM grid (formvar-carbon coated copper film, 200-400 mesh) and dried. All images for particle-size were analysed by ImageJ software.

Energy dispersive X-ray (EDX) spectroscopy measurements were recorded on a TALOS F200X STEM.

Fourier-Transform InfraRed (FT-IR) spectroscopy of nanoparticle samples was performed on a Varian 640-IR spectrometer.

Nitrogen porosimetry was performed at 77 K on a Nova porosimeter instrument in the relative pressure range 0.01 - 0.99 P/P_0. The samples were dried and degassed at 150 °C overnight .. The samples were analysed using 12 mm wide glass tubes.

Solid-state magic-angle spinning nuclear magnetic resonance (MAS NMR) spectroscopy was recorded on a Bruker Avance Neo 14.1 T spectrometer equipped with a 4 mm MAS probe. ^{29}Si NMR spectra were measured under 8 kHz MAS with a radiofrequency field amplitude of 70 kHz and referenced to the ^{13}C signal of adamantane (38.48 ppm for methylene) adjusting for the ratio of the ^{13}C:^{29}Si Larmor frequencies, in accordance with IUPAC recommendation. For each sample the ^{29}Si spin-lattice relaxation time constant, T_1, was measured for each of the ^{29}Si signals observed. Subsequently, a quantitative ^{29}Si spectrum of the sample was recorded using a recycle delay of approximately five times the longest of the T_1 values measured for that sample.

Sample	T_1 (s) (longest)	Recycle delay (s)	Number of scans
**SiO_2**	56.4	250	92
**$PhSiO_2$@SiO_2**	40.6	250	456
**RIF⊂$PhSiO_2$@SiO_2**	40.7	250	308
**$PhSiO_2$·SiO_2**	56.4	250	152
**RIF⊂$PhSiO_2$·SiO_2**	55.1	250	1468

In vitro rifampicin release experiments. For all samples, dry solid particles were immersed in the release media of DMSO (2 mg/mL in 5 mL) at room temperature. For a measurement of release, 1 mL of the particle suspension was removed and centrifuged for 5 min at 7830 rpm. The supernatant was collected and analysed by UV-vis spectrometry by monitoring drug absorbance at the wavelength maximum (λ_{max} = 480 nm, ε = 8120 $M^{-1}cm^{-1}$). The concentration of drug was calculated

by its calibration curve that determined the absorption coefficient (ε, $M^{-1}cm^{-1}$). The amount of drug (μg) in the particle (mg) could then be determined. Each experiment was repeated in triplicate.

Ultrasound-responsive triggered drug release in short intervals. To confirm the drug release from the nanoparticle structure with an ultrasonic scaler (P5 Newtron XS, Satelec, Acteon, France) with a US frequency of 29 kHz and tip 10P was used for mechanical cavitation at low power (P10, 0.27 W). The power settings relate to the dial control on the ultrasonic scaler. The ultrasonic scaler hand-held piece was positioned inside a 50 mL centrifugal tube, immersed in 5 mL release media. Subsequently, cavitation was applied using the tip of the hand-held piece. After 30 min, 1 mL of the particle suspension was removed and centrifuged for 5 min at 7830 rpm. The supernatant was collected and analysed by LCMS. The concentration of drug was calculated by its calibration curve. The amount of drug (μg) in the particle (mg) could then be determined.

Liquid chromatography mass *spectrometry (LC-MS)* analyses were carried out on a Waters Acquity H-Class Plus UPLC coupled with a Thames-Restek Raptor C18 100 mm x 2.1 mm x 1.8 μm column. Formic acid (FA) was added to both eluents. The samples were submitted as 1:1 H_2O:MeCN solutions, a two-time dilution of the sample concentration.

Time / min	Flow / μLmin^{-1}	A: H_2O, 0.1% FA / %	B: Acetonitrile, 0.1% FA / %
0.00	0.2	70	30
0.25	0.2	70	30
0.50	0.4	70	30
3.00	0.4	20	80
4.25	0.4	20	80
5.00	0.4	70	30

Mass spectrometry was performed using Waters Xevo G2-XS QToF spectrometer with ESI ionisation, spray voltage: 3 kV, sample cone: 35 V, sampling cone temperature: 100 °C, desolvation temperature 250 °C, sensitivity mode (resolution 22,000 FWHM) with selective reaction monitoring (SRM): m/z 823.4-791.4, collision energy: 20.1 V.

Fluorescence spectroscopy. Steady state emission measurements were recorded on an Edinburgh Instruments FLS1000 steady state and time-resolved spectrometer coupled to a R928 Hamamatsu photomultiplier tube in the solid state at T = 293 K. Fluoracle software was used to record the data and all spectra were corrected for photomultiplier and instrument response. Quantum yield determinations of powders were obtained using an integrating sphere using unloaded particles as a blank. Analysis of quantum yield calculations were performed with the Fluoracle software absolute quantum yield wizard. Quantum yield values are calculated as an average of three independent measurements.

Biological Studies

General procedure. Brain heart infusion (BHI, CM1135, Oxoid, Dorset, UK) broth and agar (BHI, CM1135, Oxoid, Dorset, UK) were used to culture *Staphylococcus aureus* (strain SH1000),[1] from frozen stocks stored at -80°C. The strain was a gift from Prof. Paul Williams (University of Nottingham). Bacteria from frozen stocks were streaked on BHI agar and incubated overnight (37 °C). Liquid cultures were prepared with a single colony in 10 mL BHI broth before incubating for 20 h in an orbital shaker (37 °C, 100 rpm). Phosphate buffered saline (PBS, Dulbecco A, Sigma P4417) solution was prepared by dissolving one tablet in ultrapure H_2O (200 mL) and autoclaved for sterilisation. *Statistical analysis.* All statistical analyses were performed in Origin. For biofilm viability studies, the percentages of live and dead *S. aureus*, were represented by the mean with standard deviation for each group. Statistical tests performed are described in figures.

Minimum inhibitory concentration (MIC) assay. Overnight cultures were prepared in BHI broth (10 mL) as described above. The OD_{600} of the overnight culture was adjusted to 0.001 by diluting the bacterial inoculum with fresh BHI broth. Sterile sample stock solutions were made and then diluted for the first concentration in the dilution series with 100 µL of diluted culture into a 96-well plate and incubated for 24 h under 37 °C. For controls: 1:1 ratio of bacteria to BHI broth as positive control, BHI broth was used to show no contamination (negative control) and empty well to obtain background reading of the well plate. Growth and no growth were determined visually and by measuring the OD_{600} using a microplate reader (ELx800, BIO-TEK instruments Inc.) in triple triplicates (N=9) with the Gen5 data analysis software for all measurements.

Biofilm formation model. *Staphylococcus aureus* was used to form a single species biofilm for treatment with silica nanoparticles and an ultrasonic scaler. A liquid bacterial culture in BHI broth was prepared, with a single colony in 10 mL BHI broth before incubating for 20 h in an orbital shaker (37 °C, 190 rpm). Firstly, Thermo Scientific™ Nunc™ Thermanox™ coverslips (13 mm diameter, cell culture-treated one side) were added to a 24-well plate. The overnight bacterial suspension was diluted to OD = 0.05 in fresh BHI medium and 2 mL was added to each coverslip. The 24-well plate was incubated at 37 °C for 24 h. Afterwards the medium was removed and replaced with 2 mL fresh medium. This was repeated to obtain biofilms grown for 72 h.

Biofilm viability studies and ultrasonic scaler experimental set-up. In a 12-well plate, the Thermanox coverslips were both treated with the sample solution and no cavitation or with sample solution and ultrasound and then incubated at 37 °C for 30 min. The biofilm treatment process was achieved using a Satelec P5 Newtron XS scaler with a 10P tip in all experiments. The hand piece of the scaler was fixed to a manual clamp stand and adjusted to 10 mm from the biofilm surface and the orientation of the tip was kept in a horizontal position relative to the biofilms. The biofilm coverslips were fixed in place to the surface of the 12-well plate using UV-sterilised doubled-sided tape and the wells were filled with 0.1 M PBS solution (3 mL) to completely immerse the tip surface of the scaler. The ultrasonic scaler was operated at low (P10) power for 10 s.

Live/dead staining assay. Filmtracer™ LIVE/DEAD® Biofilm Viability Kit (Invitrogen, California, USA) was used to stain the biofilms. A stock solution of the stain was prepared with SYTO® 9 stain (3 µL) and propidium iodide (3 µL) in 0.1 M PBS solution (1 mL), this was enough to stain 5 biofilms. After the biofilms had been treated, they were washed with 0.1 M PBS solution and the staining solution (~200 µL) was added gently to the biofilm. The biofilms were incubated for 20 min at room temperature and protected from light exposure with foil. The stain was removed, and biofilms were washed by rinsing gently once with 0.1 M PBS solution to remove all excess strain. To fix the biofilms, the coverslips were placed onto microscope slides (biofilm facing up) and a drop of Invitrogen™ ProLong™ Gold Antifade Mountant (ThermoFisher Scientific, Massachusetts, USA) was added before putting a 22 x 26 mm cover glass on top. The slides were left to solidify and dry for 24 h and were stored in the dark to preserve fluorescence.

Confocal laser scanning microscopy imaging. The biofilms' viability was imaged with a confocal laser scanning microscope (LSM 880 and LSM 700, Carl Zeiss GmbH, Germany). The images were obtained with the Zeiss Zen lite 2011 software. A 40x oil immersion objective (Zeiss Objective EC Plan-Neofluar 40x/1.30 Oil DIC M27, FWD = 0.21 mm) and a x100 oil immersion objective (100x/1.4 Plan-Apo (oil) DIC III) in combination with an immersion oil (Immersol™ 518F) was used. The emission/excitation wavelengths for the stains were 488 nm/<550 nm for SYTO® 9 and 555 nm/>550

nm for propidium iodide. Both the green and red channels were imaged together with an image size of 1024 x 1024 pixels. Five random locations were scanned on each biofilm sample and 2-3 Z-stacks of 10-30 µm optical thickness separated by 0.4-1.30 µm increments from the surface were obtained for each condition. Z-stacks were examined to calculate the biofilm thickness and for 3D visualisation analysis.

Image analysis of single-species biofilms. The percentage of live and dead bacteria in each image was determined from the confocal fluorescence images. An automated computation method developed and described by S. E. Mountcastle *et al.*[2] was used to evaluate the cell viability. The macro created was a method carried out using the ImageJ software (version 2.1.0). This was achieved by splitting the confocal fluorescence images into two different channels (green and red) and converted into an 8-bit image. A series of erosion, reconstruction and dilation steps had been incorporated into the script to perform on each channel (element size 3). The total bacteria were calculated by number of pixels in the image (green and red), differentiating between background noise depending on intensity values. A Gamma command (set at 1.5) was used to correct for uneven fluorescence intensities and allowed for detection of faint bacteria. Then segmentation and thresholding were performed by using the Otsu's threshold. The output was presented as white pixels, which the number of pixels corresponding to the dead (red) bacteria was calculated. This was used to determine the area of dead bacteria, followed by the total number of white pixels, which were used to calculate the total area of all bacteria. Finally, the percentage of viable cells (green) were calculated from the total area of bacteria and red bacteria. The macro is used based on the assumption that the image contains a single-species biofilm and output is a percentage of live cell area.

Colony counting method - colony forming units (CFUs). An alternative quantification method to determine bacterial survival was conducted by counting the number of colony forming units in a bacterial suspension, a well-known procedure known as the Miles and Misra method.[3] After treatment the biofilms were removed from each well and coverslip and redispersed by vortexing for 2 minutes in 0.1 M PBS solution buffer (2 mL). A serial dilution was performed using the Miles and Misra method to count the number of CFUs. 0.1 M PBS solution (180 µL) was aliquoted into each well of a 96 well plate with 20 µL of samples for the first dilution (1×10^{-1}). Subsequently, 20 µL was taken from the first dilution and added to the next well with 0.1 M PBS solution, repeating the process to 1×10^{-8} dilution. For each dilution, 20 µL aliquots were spotted onto BHI agar plates three times. This was repeated for each sample, and all plates were incubated at room temperature for 48 h. Following this, the number of CFU/mL was determined for each sample prepared in triplicate.

MTT cell viability assays. Cytotoxicity of studied nanoparticles was assessed by MTT (3-(4,5-dimethylthiazol-2-yl)-2,5-diphenyltetrazolium bromide) using H400 human epithelial cell line and THP-1 human monocyte cell lines. Before the treatments, H400 cells were seeded at 1×10^4 cells / well in 96-well plates (100 µL) and left to incubate (5 % CO_2, 37 °C, > 90 % humidity) for 24 h for attachment to occur. THP-1 cells (3×10^5 cells / well) were differentiated into macrophages by treatment with 50 ng/mL PMA (phorbol 12-myristate 13-acetate) for 24 hours. After 24 h, the media was removed and fresh media (100 µL) containing nanoparticles of different concentrations was added. The cells were incubated for 24 h (5 % CO_2, 37 °C, > 90% humidity). After 24-h treatment, the culture medium was removed from the plates. 50 µL of MTT solution (1 mg/mL) was added to each well and plates further incubated for 2 h (5 % CO_2, 37 °C, > 90 % humidity). Then, the MTT solution was removed gently to avoid loss of any newly formed purple crystals and 100 µL of DMSO (anhydrous 99%) added to each well. The plate was covered in foil and shaken on an orbital shaker for 30 min to fully dissolve the MTT formazan. The metabolic activity was determined by reading absorbance at OD_{570} using a microplate reader (ELx800, BIO-TEK instruments Inc for H400 cells, and BMG Omega for THP-1 cells).

Biofilm Viability (MTT) assay. To quantify *S. aureus* biofilm metabolic activity after treatment, we applied the MTT assay in a 24 well polystyrene plates. A stock solution of MTT reagent was prepared in 0.1 M PBS solution (0.5 mg/mL). After the biofilms had been treated, they were washed with 0.1 M PBS solution and the staining solution (~500 µL) was added gently to the biofilm. The biofilms were incubated for 3 h in a static incubator at 37 °C. The MTT solution was gently removed to avoiding loss of any newly formed purple crystals. Next, 500 µL of lysing solution (DMSO, anhydrous 99%) was added to each well to dissolve formed MTT formazan. The plate was covered in foil and shaken on an orbital shaker for 30 min to fully dissolve the MTT formazan. The metabolic activity

was determined by reading absorbance at OD_{570} using a microplate reader (ELx800, BIO-TEK instruments Inc.).

Scanning electron microscopy (SEM) imaging of biofilms. For further visualisation of bacterial biofilms, samples were prepared for SEM imaging by a fixation and dehydration procedure described by Dysktra *et al*.[4] The biofilms were rinsed three times with 0.1 M PBS solution to remove the culture medium. Biofilms were fixed using 2.5% EM grade glutaraldehyde in 0.1 M sodium cacodylate buffer, pH 7.3 for 10 min. Note the fixative must be prepared fresh but can be kept up to 1 week in the fridge. Dehydration of each specimen was performed by removing water slowly in ethanol solutions of increasing concentration, for at least 10 min in each %: 20, 30, 40, 50, 60, 70, 90, 95 (twice) and 100 % (twice). The ethanol (100 %) was completely removed and hexamethyldisilane (HMDS, Sigma-Aldrich) was added quickly to cover the surface before the sample could dry out and left to evaporate overnight in a fume cupboard. After the fixation and dehydration processes, the biofilms were prepared for imaging with the SEM. The biofilm coated coverslips were adhered onto aluminium stubs using 12 mm carbon adhesive tabs (Agar Scientific) and secured with single-sided conductive copper tape. All samples were sputter coated with gold (Quorum Emitech K550X, Kent, UK) before insertion into the SEM. The morphology and changes to the bacteria before and post treatment were examined using a Zeiss Evo MA-10 SEM (Carl Zeiss Jena GmbH, Germany) and SmartSEM software. Imaging was performed with a working distance of 10 mm and accelerating electron beam voltage of 20 kV.

Additional Nanoparticle Characterisation

Table S1. Summary of DLS sizes (number, intensity and volume and PDI), zeta-potential measurements and TEM diameters of core@shell and co-condensation nanoparticles.

Sample	DLS diameter / nm				ζ-potential / mV	TEM diameter / nm
	Number	Intensity	Volume	PDI		
**$PhSiO_2@SiO_2$**	50 ± 25	120 ± 67	150 ± 60	0.25	-32 ± 6	60 ± 19
**RIF⊂$PhSiO_2@SiO_2$**	90 ± 20	110 ± 30	100 ± 30	0.26	-46 ± 7	90 ± 25
**NR⊂$PhSiO_2@SiO_2$**	90 ± 18	100 ± 25	90 ± 25	0.39	-28 ± 8	50 ± 17
**RIF+NR⊂$PhSiO_2@SiO_2$**	160 ± 47	180 ± 42	190 ± 57	0.31	-22 ± 9	90 ± 13
**$PhSiO_2·SiO_2$**	270 ± 57	280 ± 52	290 ± 160	0.39	-30 ± 10	150 ± 15
**RIF⊂$PhSiO_2·SiO_2$**	430 ± 60	430 ± 42	440 ± 62	0.69	-27 ± 5	54 ± 6

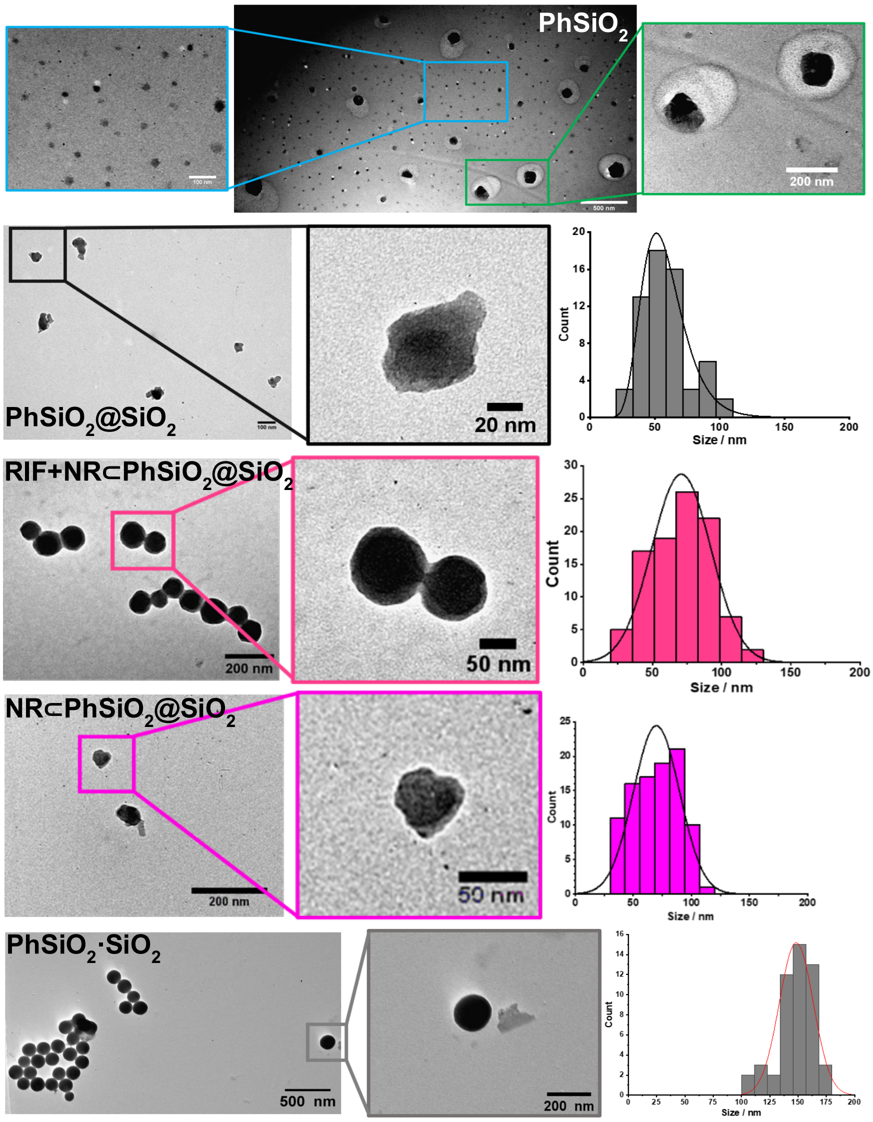

Figure S1. TEM images of a range of nanoparticles with size distribution histogram.

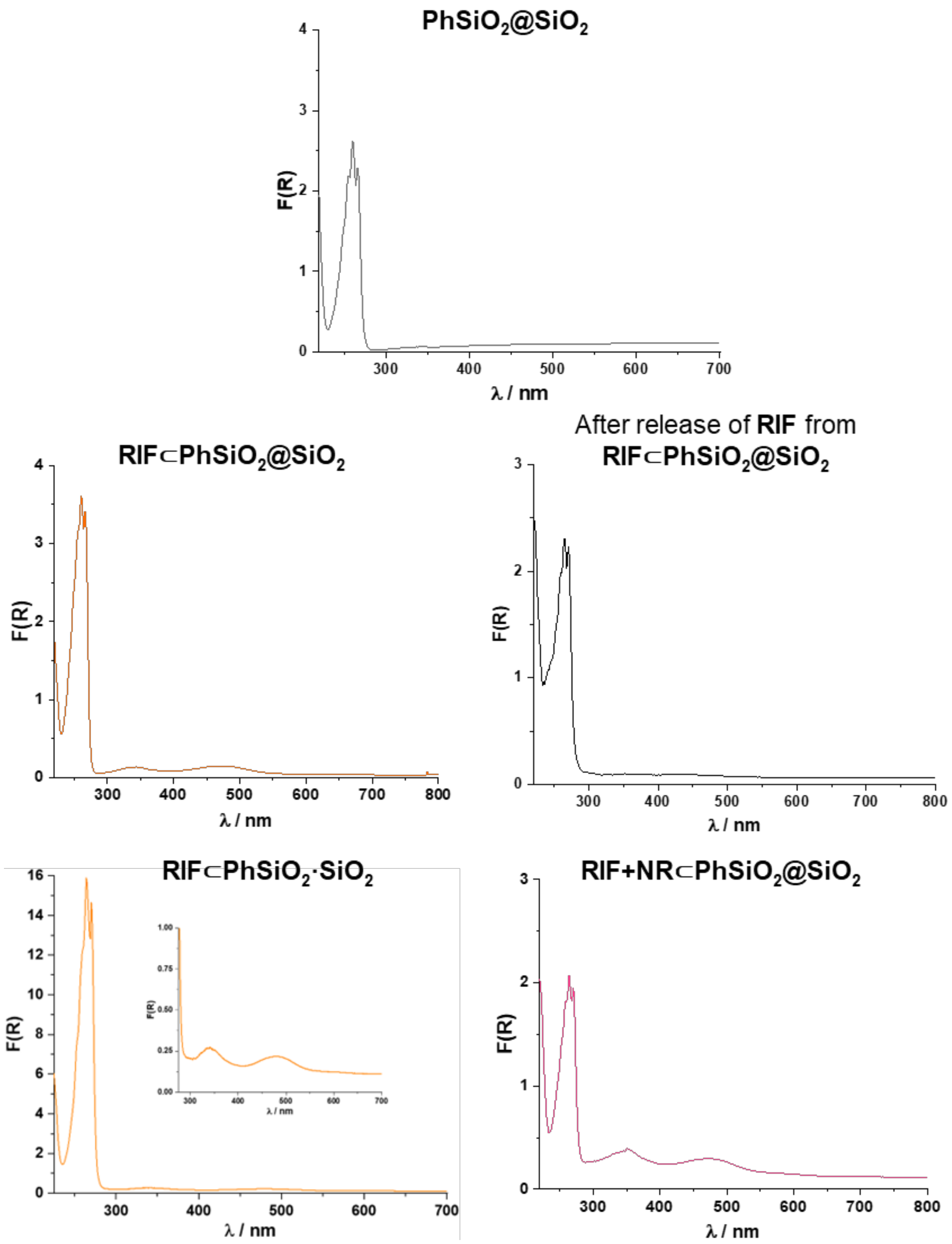

Figure S2. UV-vis spectra (measured in reflectance mode) of nanoparticles**.**

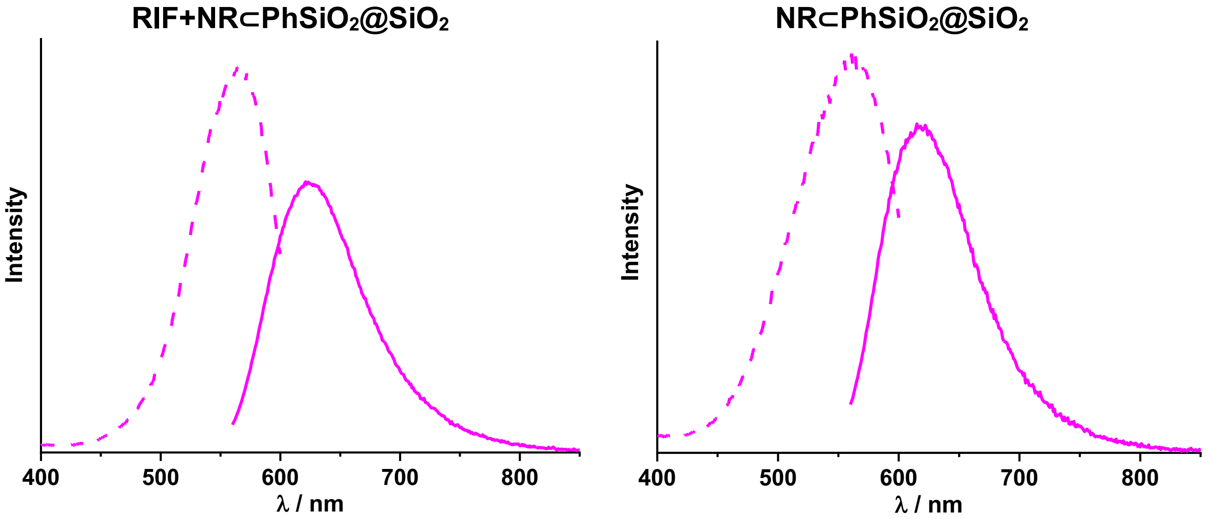

Figure S3. Excitation (λ_{em}= 610 nm, dashed line) and fluorescence (λ_{exc}= 530 nm, solid line) spectra of **RIF+NR⊂PhSiO$_2$@SiO$_2$** and **NR⊂PhSiO$_2$@SiO$_2$**.

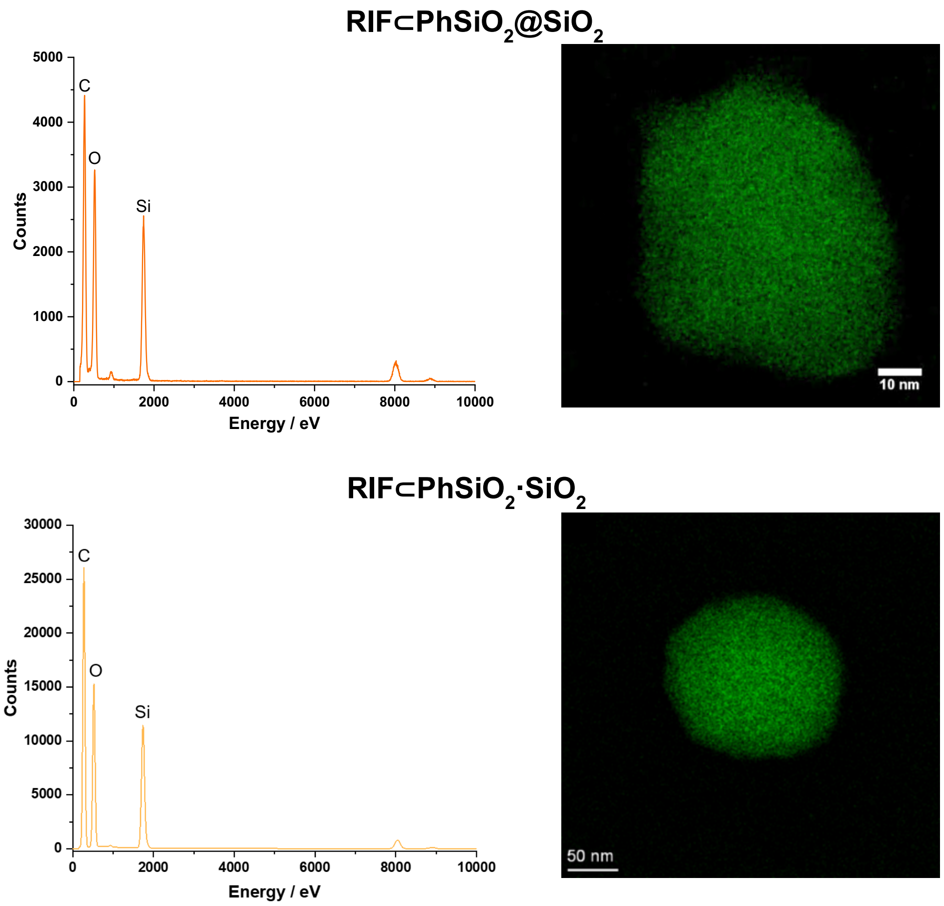

Figure S4. EDX spectra and mapping of oxygen presence in the two types of nanoparticles.

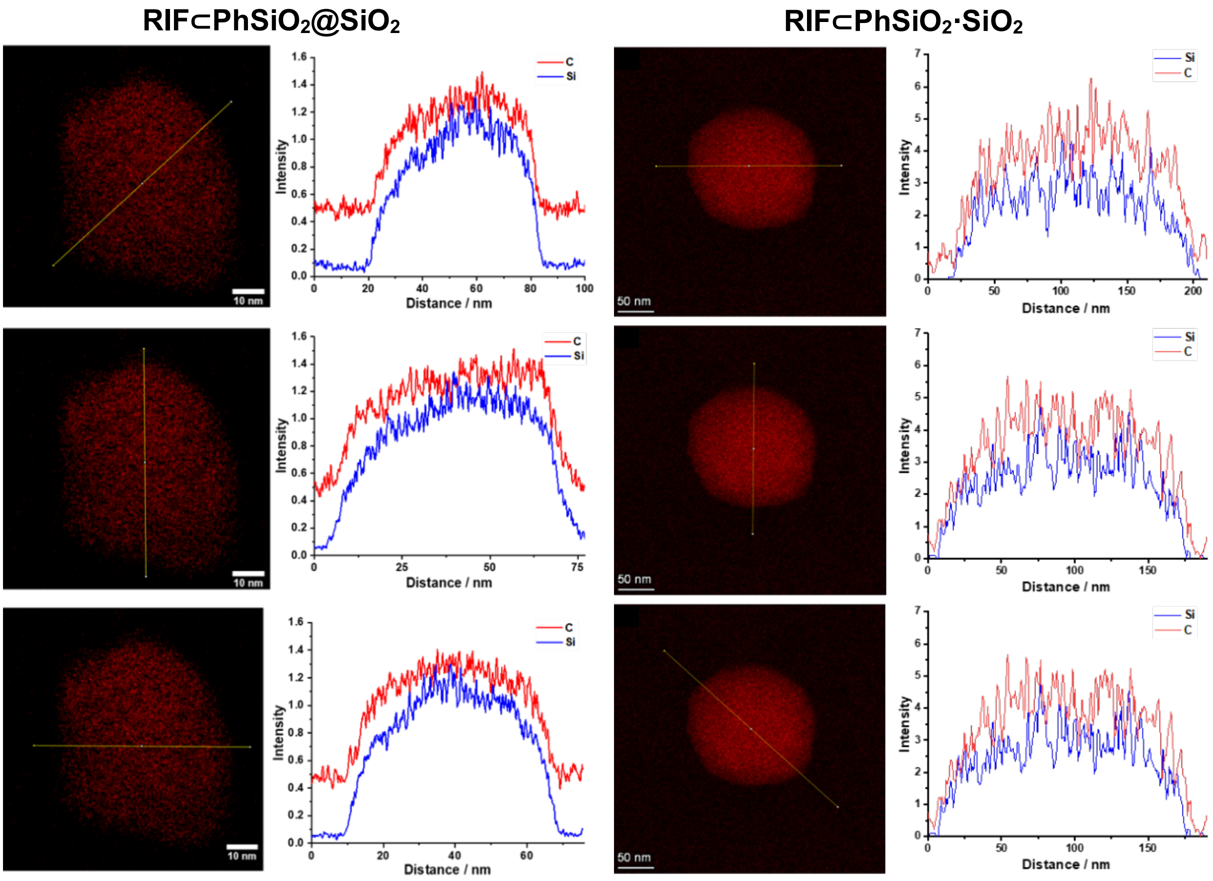

Figure S5. Intensity profiles of silicon and carbon within **RIF⊂PhSiO$_2$@SiO$_2$** (left) and **RIF⊂PhSiO$_2$·SiO$_2$** (right), measured by EDX experiments showing the cross sections used (yellow line).

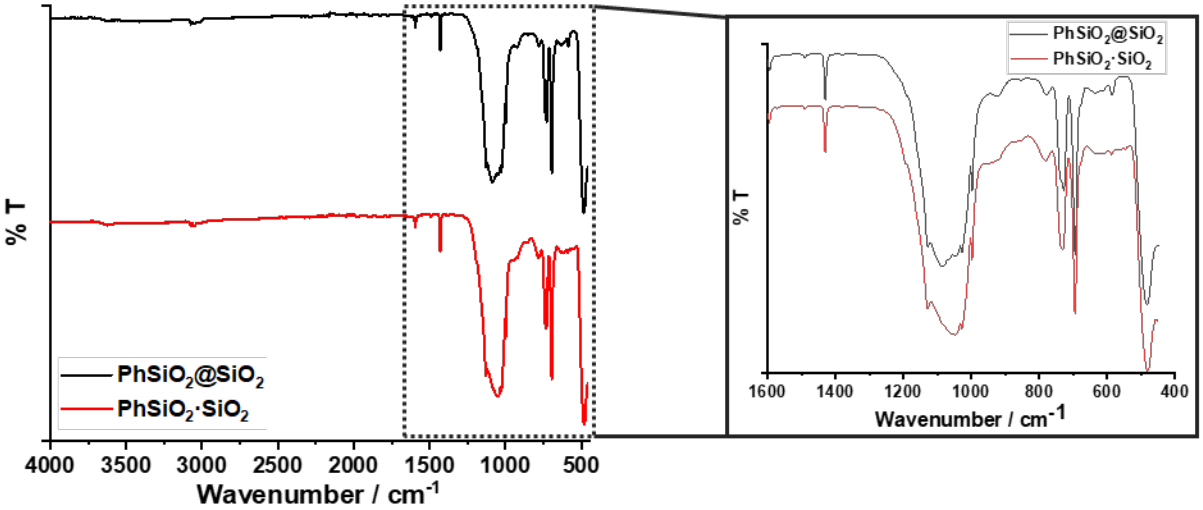

Figure S6. Stacked FT-IR spectra of **PhSiO$_2$@SiO$_2$** (black) and **PhSiO$_2$·SiO$_2$** (red), with an insert highlighting the region between 1600-450 cm^{-1}.

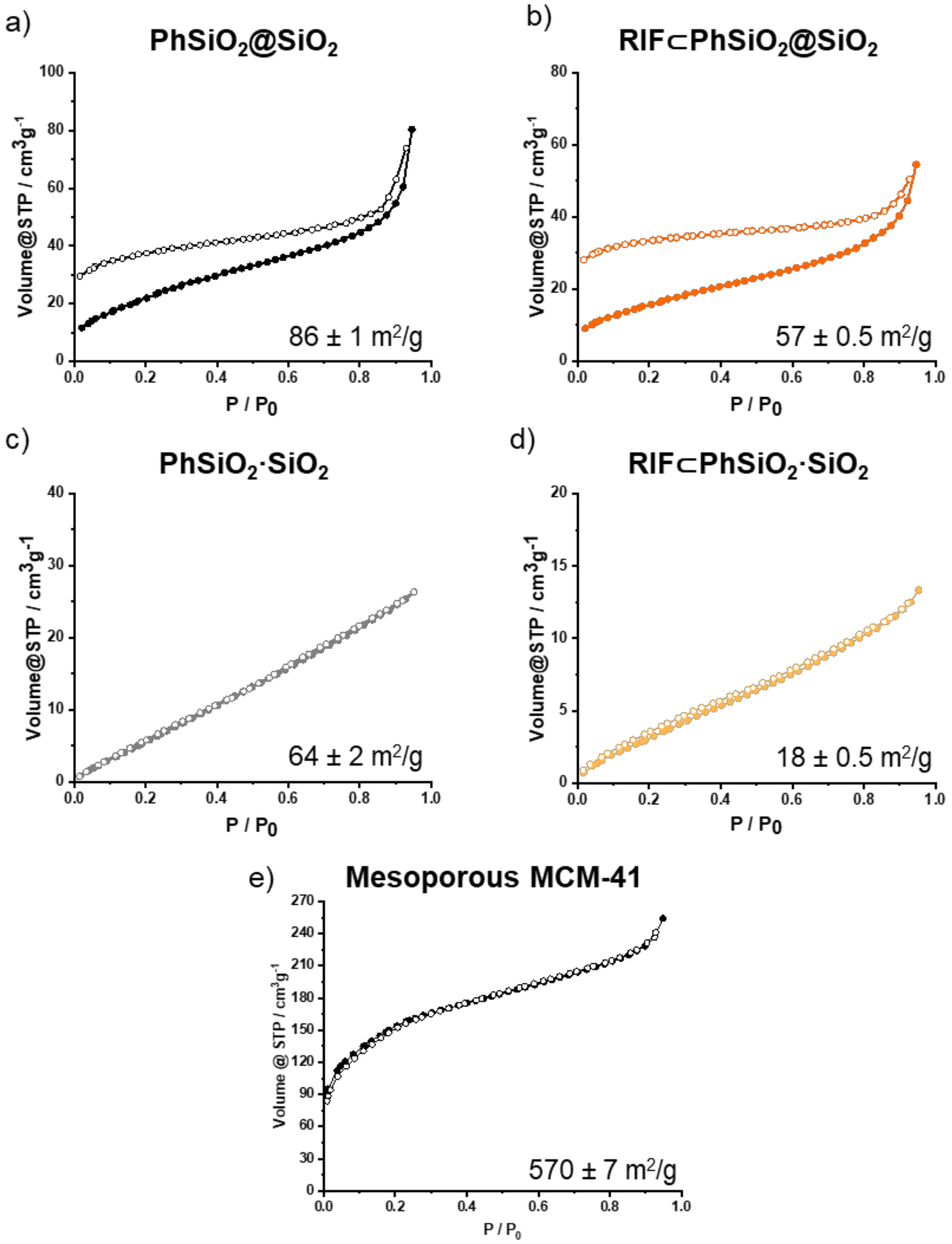

Figure S7. Nitrogen porosimetry isotherms of a) **$PhSiO_2@SiO_2$**, b) **RIF⊂$PhSiO_2@SiO_2$**, c) **$PhSiO_2·SiO_2$**, d) **RIF⊂$PhSiO_2·SiO_2$** and e) mesoporous MCM-41 nanoparticles, with BET surface areas. Solid circles indicate absorption and hollow circles indicate desorption.

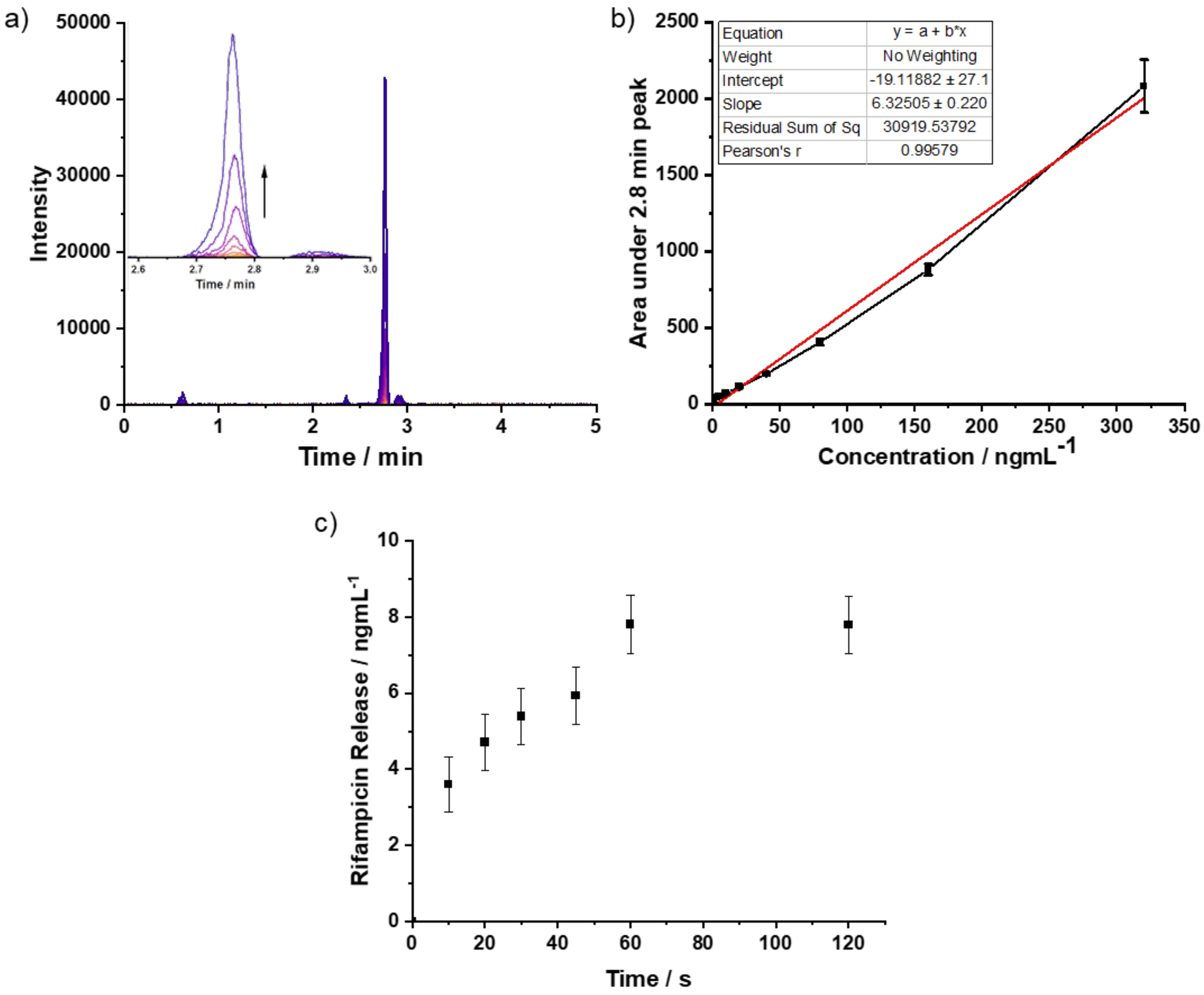

Figure S8. a) LCMS chromatogram of rifampicin and b) associated calibration curve for quantification of release of rifampicin from **RIF⊂PhSiO$_2$@SiO$_2$** and c) time-dependent release studies (0.27 W.)

Further Biological Studies

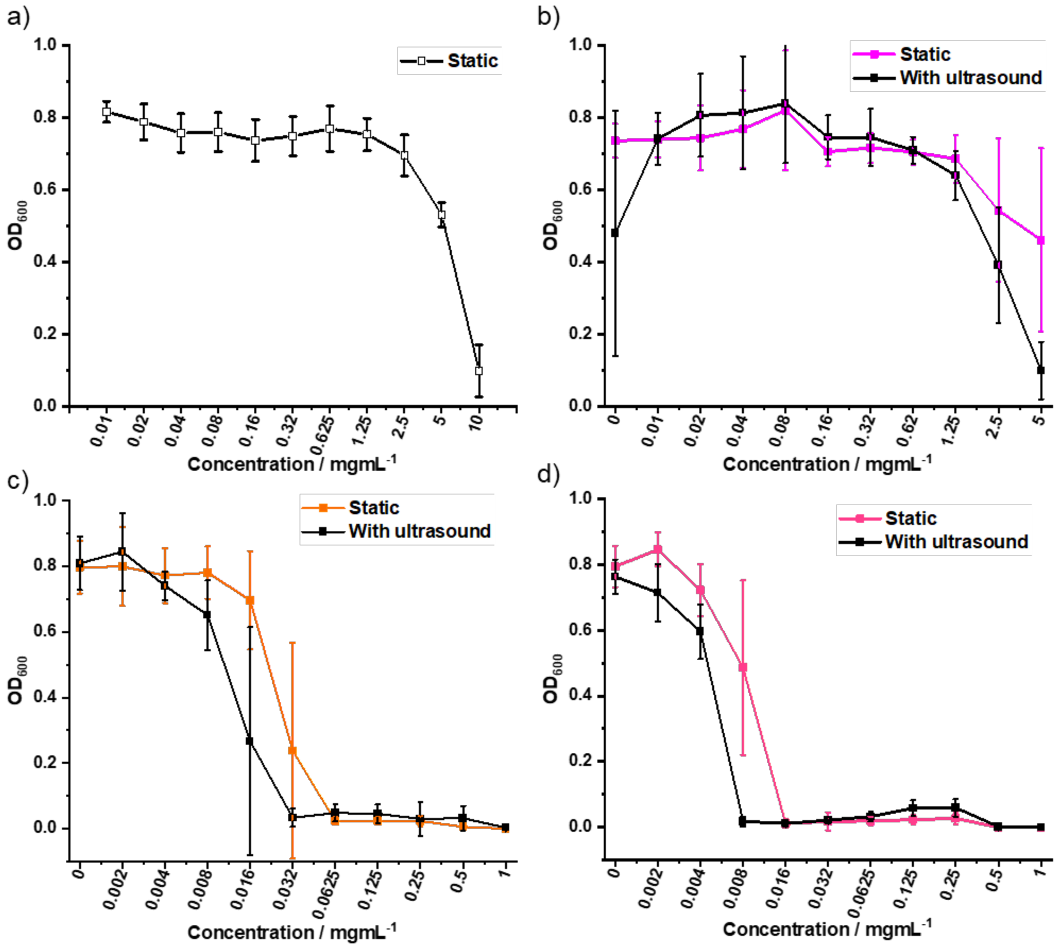

Figure S9. Minimum nanoparticle concentration for inhibition of *S. aureus* measuring OD_{600} upon treatment with a) **$PhSiO_2$@SiO_2** without ultrasound, b) **NR⊂$PhSiO_2$@SiO_2** with and without ultrasound, c) **RIF⊂$PhSiO_2$@SiO_2** with and without ultrasound and d) **RIF+NR⊂$PhSiO_2$@SiO_2** with and without ultrasound. These data are an average of triplicates and error bars indicate standard deviation.

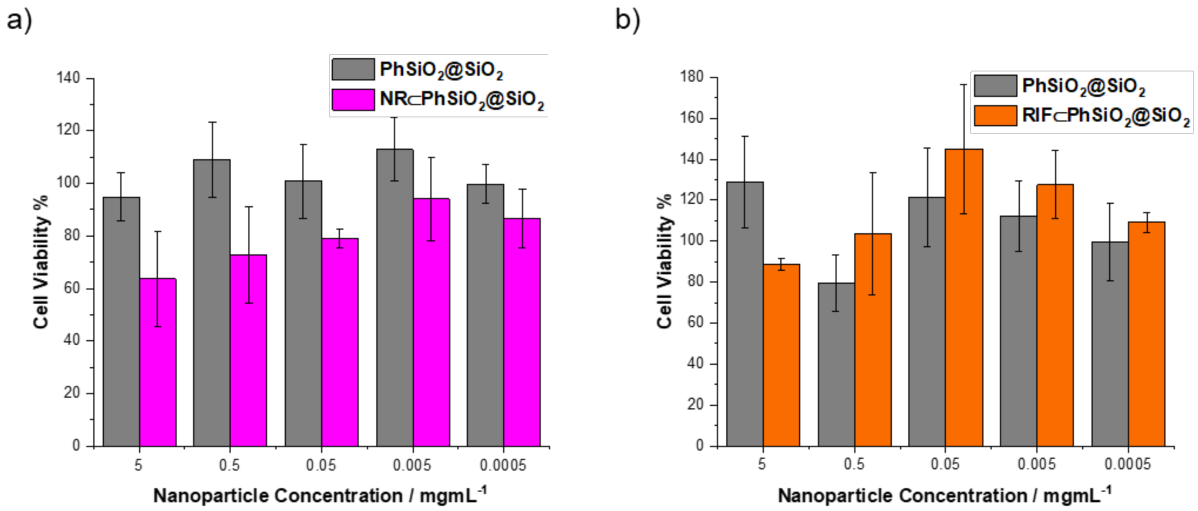

Figure S10. MTT cell viability assay of a) H400 cell line incubated with **$PhSiO_2$@SiO_2** and **NR⊂$PhSiO_2$@SiO_2** nanoparticles for 18 h and b) macrophage differentiated THP-1 cell line incubated with **$PhSiO_2$@SiO_2** and **RIF⊂$PhSiO_2$@SiO_2** nanoparticles for 24 h (n=3).

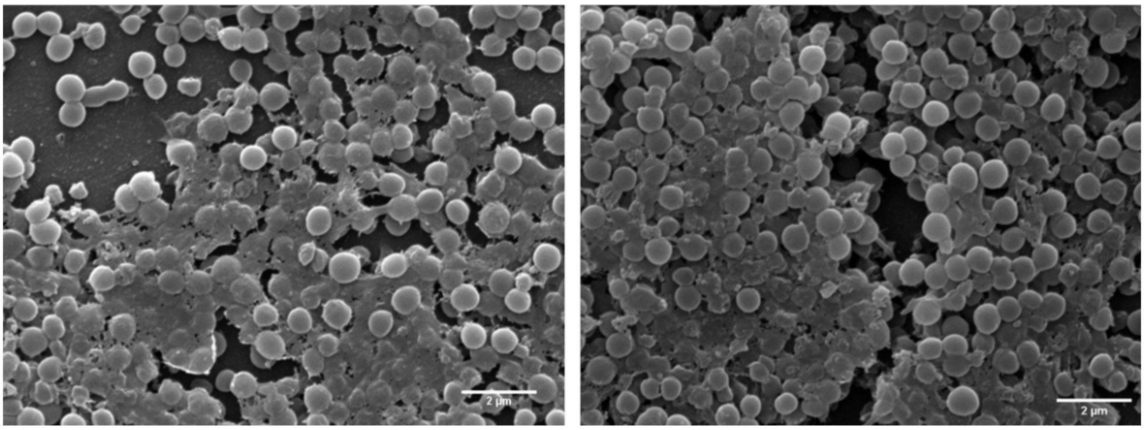

Figure S11. Further SEM images of *S. aureus* biofilms treated with **RIF⊂PhSiO$_2$@SiO$_2$** and ultrasound (52 mA, 0.27 W) at 20k times magnification. Scale bars are 2 μm (n=3).

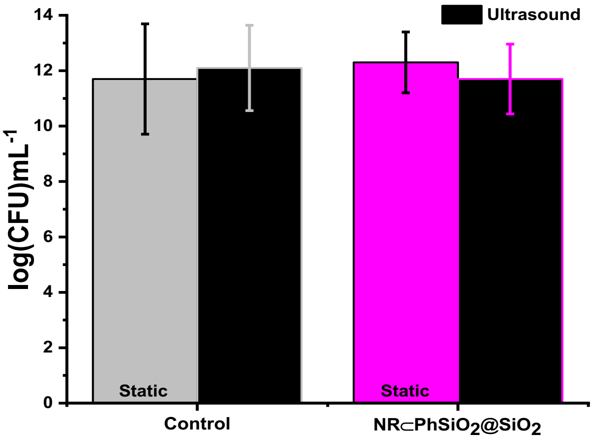

Figure S12. CFU counting of 72-h *S. aureus* biofilms treated with **NR⊂PhSiO$_2$@SiO$_2$** (1 mg/mL) with and without the application of ultrasound (0.27 W, 10 s) . Mean and standard deviation (error bars) are presented as an average of three independent experiments. Mean and standard deviation of biofilms tested for statistical significance using a two-tailed t-test (* $p < 0.5$, ** $p < 0.05$, *** $p < 0.005$).

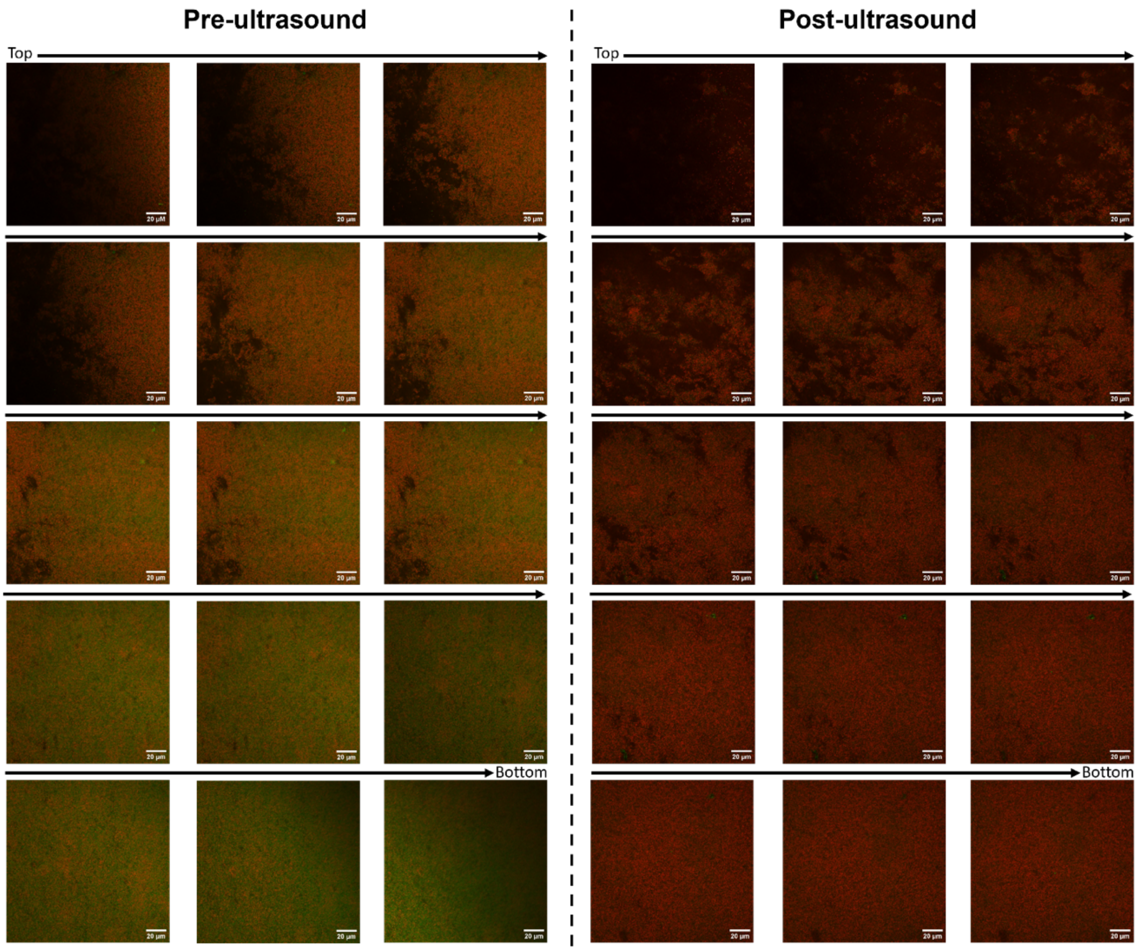

Figure S13. Individual slices of a confocal fluorescence z-stacks showing the LIVE/DEAD (STYO 9 and PI) stained 72 h grown *S. aureus* biofilm treated with **RIF⊂PhSiO$_2$@SiO$_2$** (1 mg/mL) before (left) and after ultrasound (n=3). Scale bars are 20 µm.

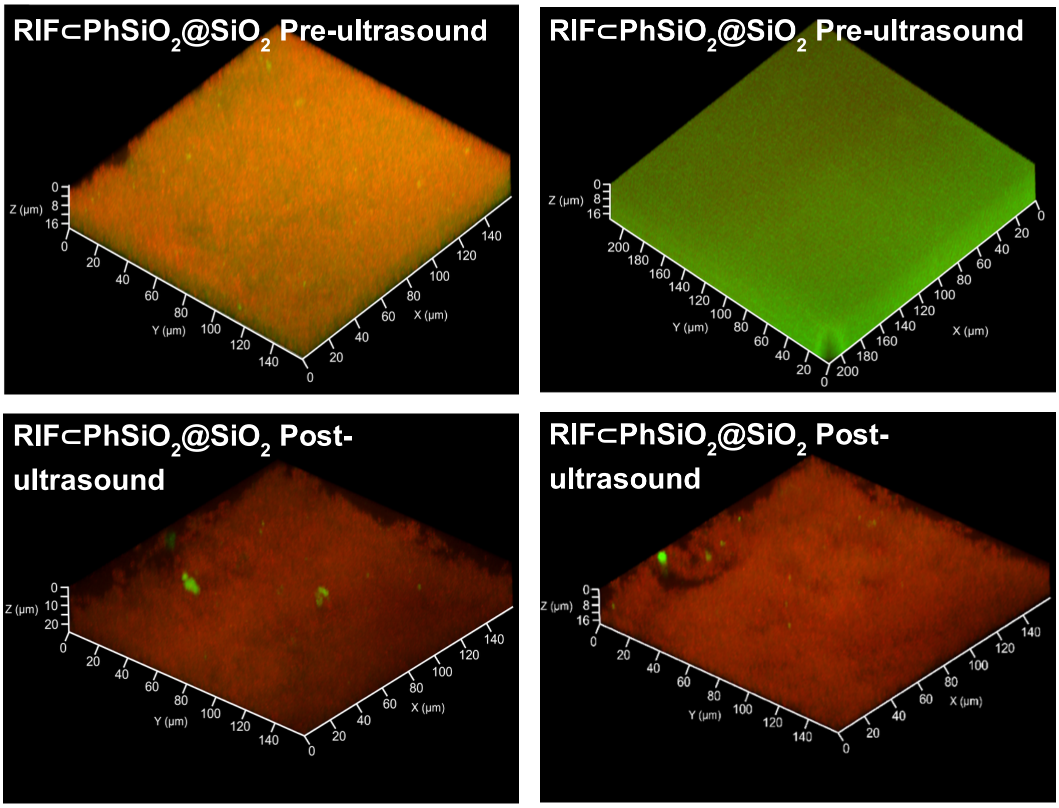

Figure S14. Further 3D confocal fluorescence images of LIVE/DEAD (STYO 9 and PI) stained 72 h grown *S. aureus* biofilms treated with **RIF⊂$PhSiO_2@SiO_2$** (1 mg/mL) pre- and post-ultrasound treatment.

References

1. O'Neill, A. J., Staphylococcus aureus SH1000 and 8325-4: Comparative Genome Sequences of Key Laboratory Strains in Staphylococcal Research. *Lett. Appl. Microbiol.* **2010,** *51* (3), 358-361.

2. Mountcastle, S. E.; Vyas, N.; Villapun, V. M.; Cox, S. C.; Jabbari, S.; Sammons, R. L.; Shelton, R. M.; Walmsley, A. D.; Kuehne, S. A., Biofilm viability checker: an open-source tool for automated biofilm viability analysis from confocal microscopy images. *npj Biofilms Microbiomes* **2021,** *7* (1), 44.

3. Miles, A. A.; Misra, S. S.; Irwin, J. O., The estimation of the bactericidal power of the blood. *Epidemiol. Infect.* **1938,** *38* (6), 732-749.

4. Dykstra, M. J.; Mann, P. C.; Elwell, M. R.; Ching, S. V., Suggested standard operating procedures (SOPs) for the preparation of electron microscopy samples for toxicology/pathology studies in a GLP environment. *Toxicol. Pathol.* **2002,** *30* (6), 735-743.
